# A review of improvements on electric vehicle battery

**DOI:** 10.1016/j.heliyon.2024.e34806

**Published:** 2024-07-25

**Authors:** Alex K. Koech, Gershom Mwandila, Francis Mulolani

**Affiliations:** a Chemical Engineering Department, School of Mines and Mineral Sciences, Copperbelt University, Zambia; b Copperbelt University Africa Centre of Excellence (CBU-ACESM), Zambia; c Electrical Engineering Department, School of Engineering, Copperbelt University, Zambia

**Keywords:** Fast-charging lithium-ion batteries, Solid electrolyte interphase, Lithium plating, Thermal management, Battery management systems

## Abstract

The development of efficient and high-performance electric vehicle (EV) batteries relies on improving various components, such as the anode and cathode electrodes, separators, and electrolytes. This review paper offers an elaborate overview of different materials for these components, emphasizing their respective contributions to the improvement of EV battery performance. Carbon-based materials, metal composites, and polymer nanocomposites are explored for the anode, offering high energy density and capacity. However, they are noted to be susceptible to Li plating. Unique structures, such as Titanium niobium oxide (TiNb_2_O_7_), offer high theoretical capacity, quick Li^+^ intercalation, and an extended lifecycle. Meanwhile, molybdenum disulfide (MoS_2_), with 2D and 3D structures, exhibits high reversible specific capacity, outstanding rate performance, and cyclic stability, showing promising properties as anode material. For cathodes, lithium-iron phosphate (LFP), lithium-cobalt oxide (LCO), lithium-nickel-cobalt-aluminum oxide (NCA), lithium-nickel-manganese-cobalt oxide (NMC), and cobalt-free lithium-nickel-manganese oxide (NMO) are considered, offering specific energy and capacity advantages. For instance, LFP cathode electrodes show good thermal stability, good electrochemical performance, and long lifespan, while NMC exhibits high specific energy, relatively high capacity, and cost savings. NCA has a high specific energy, decent specific power, large capacity, and a long lifecycle. NMO shows excellent rate capability, cyclic stability, and cost-effectiveness but with limited cycle performance. Separator innovations, including polyolefin materials, nanofiber separators, graphene-based composites, and ceramic-polymer composites, are analyzed for use as separators, considering mechanical strength, porosity, wettability with the electrolyte, electrolytic absorption, cycling efficiency, and ionic conductivity. The electrolyte comprises lithium salts such as lithium tetrafluoroborate (LiBF_4_), lithium hexafluorophosphate (LiPF_6_), and other salts dissolved in carbonate solvents. This improves energy density, capacity, and cycling stability and provides high ion mobility and resistance to decomposition. By examining the existing literature, this review also explores research on the solid electrolyte interface (SEI) and lithium plating, providing valuable insights into understanding and mitigating these critical issues. Despite the progress, limitations such as practical implementation challenges, potential cost implications, and the need for further research on scale-up feasibility and long-term durability are acknowledged. These efforts to enhance the electrochemical characteristics of key battery parameters—positive and negative electrodes, separators, and electrolytes—aim to improve capacity, specific energy density, and overall energy density. These continuous endeavours strive for faster charging of EV batteries and longer travel ranges, contributing to the ongoing evolution of EV energy storage systems. Thus, this review paper not only explores remarkable strides in EV battery technology but also underscores the imperative of addressing challenges and propelling future research for sustainable and high-performance electric vehicle energy storage systems.

## Nomenclature

kWh: kilowatt-hour
m^2^/s: square meters per second (diffusion coefficient)
A/m^2^/mol: Ampere per square meter per mole
ESVR: electrode surface/volume ratio
°C: degrees Celsius
M: Molarity
V: volts
mV: millivolts
mA h g⁻^1^: milliampere-hours per gram
g cm⁻³: gram per cubic centimeter
C: Coulomb
2D: two dimensional
3D: three dimensional
mAh cm⁻^2^: milliampere-hours per square centimeter
mg cm⁻^2^: milligrams per square centimeter

## 1 Introduction

While automakers race to include electrified options in their ranges, customer adoption of electricvehicles (EVs), especially those powered purely by an EV battery and not those hybridized with internal combustion engines (ICEs), still needs to improve. Concerns about costs, range anxiety and the longer duration of charging in comparison to the quick refuelling process of conventional petrol vehicles are frequently cited as key factors impeding the broader acceptance of EVs [1,2]. It is important to note that there are already existing EV batteries running efficiently. However, their wide use has been hindered by safety concerns particularly related to explosions at high operating temperatures, affordability issues associated with the cost of an EV battery, and functional efficiency challenges related to charge/recharge and discharge times [3]. Electric and hybrid vehicles are becoming widely used as researchers and users adopt them because of ever-increasing fossil fuel prices and as an attempt to lessen the effect of the use of fossil fuels on the environment. The continuous use of these fossil fuels has led to severe climatic changes, thus affecting the Earth's ecosystems and causing human and environmental health issues [4]. Nevertheless, the world requires energy, particularly in the transportation sector, as exemplified by electric vehicles (EVs) – a promising technology for achieving a sustainable future. With their minimal to zero carbon emissions, EVs consume less energy and emit zero tailpipe greenhouse gases (GHG), thereby contributing to the promotion of social and economic development and the enhancement of the quality of life [5,6]. Such energy is significantly required in developing countries [7].

EV batteries are gaining popularity, and they are expected to replace conventional fossil fuels to power vehicles because of their capacity for effective energy storage and their positive impact on the environment, as they possess significant potential [8]. EV batteries are becoming widely researched for powering vehicles due to their intrinsic benefits over other battery systems. For instance, they have a higher voltage and specific capacity, enabling longer driving ranges on a single charge. Additionally, they exhibit high energy density, enabling compact and lightweight battery packs [9]. Unlike conventional battery technologies, EV batteries do not suffer from memory loss, ensuring consistent performance over time. They also demonstrate superior cycling performance, allowing for a greater number of charge and discharge cycles before capacity degradation [10]. Moreover, EV batteries are known for their high efficiency, converting a larger portion of stored energy into actual propulsion. They experience minimal self-discharge, meaning they can retain their charge for extended periods without significant loss. Finally, they operate effectively across a wide range of temperatures, making them suitable for diverse environmental conditions [[11], [12], [13]]. These features make technology development for EV battery applications critical [14]. Nevertheless, it is worth mentioning that the performance of EV batteries varies depending on the other metal components with which they are made, such as nickel, manganese, cobalt, iron, aluminium, and titanate [[15], [16], [17]].

As the demand for EVs continues to rise annually, the transportation sector is undergoing a swift and significant transformation, driven by continuous technological advancements in battery designs and technology [18,19]. This trend is expected to persist, with the anticipation of a gradual and swift phase-out of conventional fossil fuel-based vehicles worldwide by the automotive industry. This shift signals a transition toward less carbon-intensive technologies, including fully battery-electric as well as hybrid-electric vehicles [20]. Moreover, the productive development of EVs relies on the improvement of global values, comprehensive frameworks, related peripherals, and user-friendly programming software [21]. Achieving cost-effectiveness in the EV market involves addressing various challenges, one of which is the comparatively higher cost of energy supplied from EV batteries compared to petroleum-based fuel. This is often attributed to the expense associated with EV battery production technologies, which rely on costly manufacturing processes. To address this, researchers and manufacturers are actively focused on the identification and utilization of low-cost raw materials to reduce production expenses [22]. Efficient battery charging is a crucial requirement, necessitating the development of faster charging solutions to minimize downtime associated with longer charging durations [23]. Additionally, there is the challenge of limited range per single charge (the range capability of an EV), requiring improvement to extend the distance an EV can travel before requiring a recharge [9]. Further considerations include effective management of battery costs, ensuring flexible charging stations, fostering innovation in EV technology systems, facilitating EV sharing, addressing environmental concerns, ensuring safety and reliability, and addressing broader impacts related to EV adoption and policy development [24,25]. These challenges are pivotal not only for improving the technology but also for sustaining the growing market demand for EVs, which is highlighted by recent trends.

**Fig. 1:**
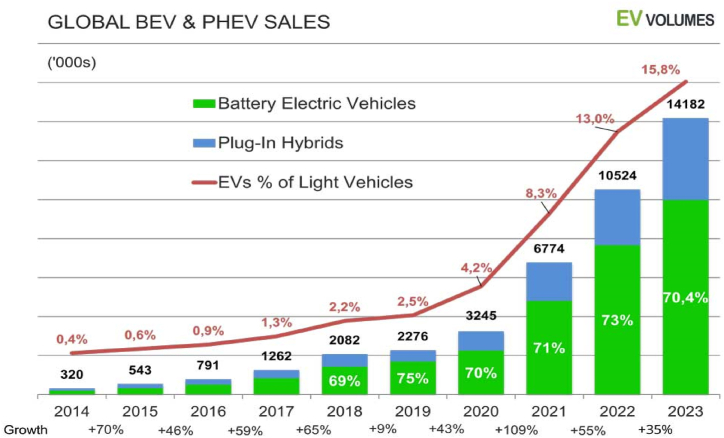
Global EV sales growth for Battery Electric Vehicles (BEVs) and Plug-in Hybrid Electric Vehicles (PHEVs) from 2014 to 2023. Adapted from Irle, 2024 [27].

Reflecting this demand, the unwavering global effort to reduce carbon emissions in transportation has led to a sustained increase in the markets for EVs and their batteries, with production and sales continuing to surge [26]. In 2023, global sales of EVs, including both Battery Electric Vehicles (BEVs) and Plug-in Hybrid Electric Vehicles (PHEVs), exhibited significant growth, as depicted in Fig. 1. Throughout the year, total EV deliveries reached 14.2 million units, marking an increase of more than 35 % over the 2022 figures [27]. However, this number represents a significant slowdown from the 54.2 % growth rate in 2022 [28,29]. Of the 14.2 million EVs sold in 2023, 10 million were BEVs, while the remaining 4.2 million consisted of PHEVs, Range Extender Electric Vehicles (REEVs), and Fuel Cell Vehicles (FCVs) [27,29].

**Fig. 2:**
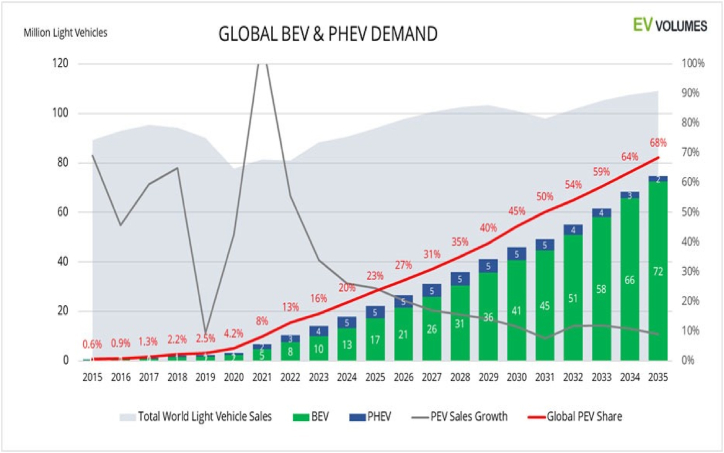
Expected and Forecasted growth in global EV sales for battery electric vehicles through to the year 2035 [31].

Global sales of EVs are forecast to increase significantly, from 10.5 million units in 2022 to over 31 million units by 2027, representing a nearly threefold increase. Projections further indicate that sales will more than double from the 2027 levels, reaching over 74.5 million units by 2035 (Fig. 2) [28,30]. Despite the substantial growth in EV sales, they will still represent a minority within the global vehicle fleet due to the large existing base of ICE vehicles. With an estimated 1.33 billion light vehicles currently in circulation, it is anticipated that by the end of 2030, EVs will constitute only 15 % of the global vehicle fleet, assuming normal vehicle scrappage rates. This proportion is expected to rise to approximately 30 % by 2035 and to achieve a 50 % share by 2042 as the global transition towards EVs progresses [30,31]. Despite these promising growth projections, the technological advancement of EVs remains critical to their broader adoption and effectiveness within the global vehicle fleet.

As a measure of this technological advancement, EV efficiency can be quantified in kilowatt-hours (kWh) of electricity it consumes per 100 miles (161 km), which is comparable to a gasoline-powered car's miles per litre statistics (although a lower kWh/100-mile rate is preferred) [32]. Wang et al. (2015) defined EV battery efficiency as the ratio of the energy required to charge a battery to the available energy during discharge [33]. EVs vary in efficiency or how far they can travel on the same amount of electricity [32]. The present fleet of small-sized battery-powered EVs, which do not employ any combustion engine as hybrids do, demonstrate a range capability that varies from 150 to slightly above 200 km. In theory, the travel range of EVs could be increased by doubling or tripling the mass of the EV batteries. However, this approach would result in prohibitively expensive EVs.

Moreover, increasing their mass would necessitate more robust car frames since batteries are inherently heavy. However, most EVs are designed to be small and light, suitable for use in city traffic, rather than large and heavy [9,34]. The ability to achieve longer EV travel ranges greatly depends on the adoption of new material systems, consideration of their energy density, fine-tuning of the lithium battery structure (through changes in the chemical system of the battery), and improvements in manufacturing capabilities—the main focus of research and development (R&D) [35].

The charging time of an EV battery cell is influenced by several factors, including the battery's chemistry, the charging infrastructure, and the battery's condition [4]. Unlike lead-acid batteries, EV batteries can be ‘fast charged’ to 100 % capacity in minutes without requiring an absorption phase to store the remaining 20 % [36]. EV batteries, commonly used in electric vehicles (EVs)*,* exhibit a charging time that typically extends over several hours (between 30 and 480 min) when compared to the few minutes that are required for replenishing the hydrogen-rich fuel or gasoline in ICE fuel tanks, although this charging duration can vary with the charging methodology employed and is usually a source of frustration for EV users [37,38]*.* Fast charging techniques have the potential to considerably decrease charging duration, although they may also lead to a reduction in the overall lifespan (battery life) of the battery over time. When a sufficient quantity of charging amperage is available, it is possible to achieve a full charge on an EV battery through fast charging in approximately 20–30 min, with the possibility of even faster charging using a few highly efficient charging units, which is a highly sought-after feature by EV users [36,39]*.* The charging of larger batteries requires a higher power input (typically more kW) and longer charging times due to their greater energy storage capacity when compared to smaller batteries. Therefore, charging times for commercial vehicles such as delivery trucks, buses, trains, and aeroplanes become significantly longer because charging stations still need to be adapted for larger batteries [39,40].

The discharge time, which refers to the time taken for an EV battery to power a vehicle before recharging, is influenced by factors such as battery chemistry, temperature, and driving conditions [41]. EV batteries have the ability to provide a range of 160–480 km on a single charge, depending on the size and capacity of the battery. However, over time, the battery's capacity can degrade, resulting in reduced discharge time [42]. To optimize the efficiency of an EV battery cell, striking a balance between charging time and discharge time is essential. Moreover, practising proper battery maintenance, such as avoiding extreme temperatures and preventing complete battery discharge, can help ensure efficient operation, longevity, and safety [43].

Since their commercialization in 1991, rechargeable Li-ion batteries have emerged as the most reliable and indispensable energy storage devices, dominating both markets and research landscapes [44]. Remarkably, the properties of Li-ion batteries underwent significant transformation following their market introduction: over the subsequent three decades, their volumetric energy density increased threefold while their cost dropped tenfold [44,45]. A Li-ion battery utilizes the reversible intercalation of lithium ions into electronically conducting solids to store energy, distinguishing itself from other rechargeable batteries. It offers greater potential for use in portable devices and EVs due to its higher specific energy, energy density, efficiency, cycle life, calendar life, and absence of memory effect [46]. This impressive advancement sets the stage for ongoing research aimed at further enhancing Li-ion battery technology. As researchers continue to push the boundaries, the focus has increasingly shifted toward optimizing battery components to meet the growing demands of EV performance and longevity.

The review paper highlights the imperative of optimizing EV battery components for enhanced performance, with particular emphasis on the need to address challenges and explore innovative solutions. While recognizing advancements in diverse materials for anode electrode improvement, such as carbon-based materials and metal composites, the discussion stresses the ongoing efforts to mitigate Li plating susceptibility through the use of novel structures like TiNb_2_O_7_ anodes, indicating a continuous pursuit of better technologies. The exploration of cathode electrode materials acknowledges their unique advantages but also underscores the ongoing search for emerging materials that could revolutionize Li-ion battery technology. For the separator and electrolyte categories, the discussion provides a comprehensive overview, and the ongoing efforts to incorporate recent breakthroughs aim at offering better ways of achieving improved performance. The discourse on SEI layer stability and the challenges associated with metallic Li anodes not only recognizes current limitations but also underscores the ongoing quest for alternative technologies aimed at enhancing Li-ion battery efficiency. In conclusion, integrating recent research findings that focus on cathode, anode, separator, and electrolyte components and addressing specific challenges is seen as a pathway to uncovering better technologies for advancing Li-ion batteries towards higher performance, reliability, and safety.

**Fig. 3:**
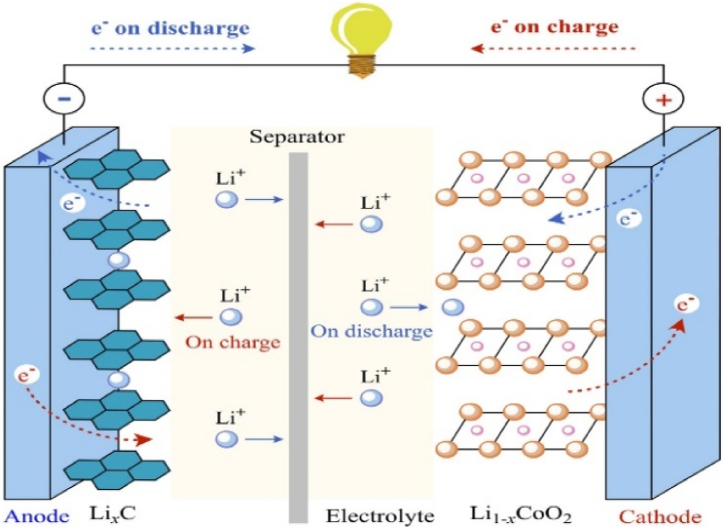
Schematic of a typical lithium-ion battery cell [18].

EV batteries are composed of multiple layers of materials that work together to store and release energy during charging and discharging cycles efficiently**.** At the atomic level, several chemical reactions take place within these layers to facilitate the flow of electrons and ions during charging and discharging [4]. The key components of an EV battery include the cathode, separator, and electrolyte, as illustrated in Fig. 3 [13], with most enhancements focused on these fundamental elements.

## 2 Performance impact of electrodes' and electrolytes' physical and chemical properties on Li-ion batteries

Several key properties significantly influence the performance of Li-ion batteries. These include lithium diffusivity within the active electrode material, the electrical conductivity of the electrodes and the electrolyte, the reaction rate constant at the electrode's active sites, and the surface area of various electrode materials [47,48]. Together, these factors impact ionic transfer, the resistance of electrodes and electrolytes, and the rate of surface reactions at the electrode-electrolyte interface. For instance, the rate at which lithium diffuses through the active materials in a battery can significantly slow the charging process, often resulting in higher overpotentials [48,49]. Moreover, the charging rate is also limited by concentration polarization caused by the sluggish transport of lithium ions through the electrolyte phase within the porous electrodes. Understanding this phenomenon is critical for optimizing the design of Li-ion batteries, as highlighted by Weiss et al., 2021 [49].

Chabot et al. (2013) [50] investigated critical factors influencing Li-ion battery performance, encompassing lithium diffusivity, electrode electrical conductivity, and reaction rate constants at active electrode sites. They found that lithium diffusivity has a lesser impact on cells with thick electrodes or high active material volume fractions. Active materials with lithium diffusivity less than 1 × 10^−14^ m^2^/sare not recommended for the positive electrode of cells with thin electrodes; hence, for optimal performance, lithium diffusivity in positive electrodes must exceed 1 × 10^−14^ m^2^/s, while negative electrodes should maintain values equal to 3.9 × 10^−14^ m^2^/s [50,51]. Enhancing lithium diffusivity in negative-electrode materials by one order of magnitude increases battery-specific energy and power density by around 11 %. For cell design, active materials with lithium diffusivities less than 3.9 × 10^−14^ m^2^/s are not recommended. Electrolyte salt concentration, however, has a negligible impact on this diffusivity's effect on cell performance [50,52,53].

Conductive additives play a very important role in the performance of Li-ion batteries by reducing the internal resistance within the electrode, improving electrolyte adsorption characteristics, and hindering electrode polarization during high-current-density charging and discharging cycles, despite constituting only a small mass percentage in both the cathode and anode [54]. Commonly utilized conductive additives in Li-ion batteries include carbon black, conductive graphite, carbon nanotubes, vapour-grown carbon fibres, and graphene [55]. Chabot et al. (2013) [50] also investigated the impact of physical and chemical properties of electrodes on Li-ion battery performance, revealing that a reduction in measured electrical conductivity to less than 0.38 S/m results in a significant decline in cell performance, particularly evident at moderate discharge rates. To mitigate this, a sufficient amount of conductive additive materials is necessary for the positive electrode [56]. However, the excessive addition of conductive material does not enhance the performance of the base cell, as the optimal conductivity level has been reached. The impact of electrical conductivity is more pronounced in cells with thicker electrodes (owing to the longer electron migration paths), low volumes of active material, or lower electrolyte salt concentrations. Conversely, varying conductivity in negative electrodes from 1 to 10^4^ S/m has minimal effect on cell performance [50]. Although conductive additives are essential for negative electrode fabrication, their quantity can be minimized, as they improve contact surface area among electrode particles, facilitating electron migration within the electrode [57].

Enhancing the reaction rate constants at the active sites of either positive or negative electrodes significantly impacts the performance of Li-ion batteries [51]. Specifically, increasing these constants to up to 2 × 10^−5^ A/m^2.5^/mol^1.5^ can improve cell performance by approximately 3–4%. This enhancement is crucial, as underestimations of reaction rates often lead to prediction errors in modelling battery behaviour, which can affect the design and optimization of battery systems. A 4 % improvement in cell performance is achieved by increasing the reaction rate constant at the active sites of either electrode by one order of magnitude [50,51].

Surface area enhancement through the adoption of micro- and nano-structured electrodes presents a promising technique to enhance Li-ion battery performance, as it increases the efficiency of ion exchange between the electrode and electrolyte. This enhancement in performance levels off once the electrode surface/volume ratio (ESVR) reaches a specific threshold and depends on the characteristics of the battery and its materials [58]. Additionally, pore structure has been found to correlate with both the surface area and pore wall thickness of the electrode material [59]. A larger specific surface area can effectively reduce the current density per unit surface area, thereby mitigating polarization at the interface and facilitating ion transfer [60].

Optimizing lithium diffusivity, electrical conductivity, and reaction rate constants at active sites is critical for improving the performance characteristics of Li-ion batteries, including their energy density, power density, cycling stability, and overall efficiency; researchers and engineers continue to explore various strategies, such as material design, nanostructuring, and electrode engineering, to enhance these key parameters and develop next-generation Li-ion battery technologies.

## 3 The electrolyte chemistries

Li et al. (2016), Kalpana and Dhoble (2021), as well as Schaefer (2012), have separately emphasized the significance of the electrolyte in determining the performance and efficiency of EV batteries. They stated that Electrolytes facilitate the movement of ions between the cathode and anode. This electrolyte typically consists of a metal salt, including lithium tetrafluoroborate (LiBF_4_), lithium hexafluorophosphate (LiPF_6_), lithium hexafluoroarsenate (LiAsF_6_) monohydrate, lithium perchlorate (LiClO_4_), lithium triflate (LiCF_3_SO_3_), and lithium trifluoromethanesulfonimide (LiN(CF_3_SO_2_)_2_), dissolved in carbonate solvents such as ethylene carbonate (EC), propylene carbonate (PC), or dimethyl carbonate (DMC) [[61], [62], [63]]. During the charging and discharging processes, the organic solvent, which functions as the electrolyte, serves as a medium for dissolving and transporting lithium ions between the anode and cathode. Since pure metals are highly reactive, non-aqueous electrolytes are commonly used in EV batteries [62]. For example, pure lithium reacts vigorously with water, resulting in the production of hydrogen gas and lithium hydroxide (LiOH). The electrolyte is thus designed to be stable and non-reactive with other cell components, including cell separators, electrode substrates (anode and cathode), and cell packaging materials [63]. It should also demonstrate effective ionic conductivity and electronic insulating properties, ensuring swift ion transfer without self-discharge. Furthermore, the electrolyte must possess good electrochemical stability to prevent degradation within the designated working potentials [64]. The choice of the electrolyte can significantly impact the battery's performance, influencing the speed at which metal ions move, with some electrolytes exhibiting higher ionic conductivity than others [65].

These characteristics, while effective, also pose limitations that today's market demands and safety concerns are challenging, prompting researchers to seek new solutions. Recent market demands for Li-ion batteries underscore the necessity for improved energy, power density, and safety, driving research into new organic solvents and lithium salts. Enhancing capacity and voltage stand out as primary strategies, highlighting the crucial need for high-voltage electrolytes due to the limited voltage stability of conventional carbonate solvents [66].

To address these emerging needs, recent studies have focused on the following: Yang, Ravdel, and Lucht (2010) [67] investigated the reaction of a common electrolyte (1 M LiPF_6_ in EC/DMC/DEC, 1:1:1) with the lithium-nickel-manganese oxide (LiNi_0.5_Mn_1.5_O_4_ -LNMO) electrode, revealing instability above 4.5 V during charging. Additionally, examination of a typical electrolyte (1.2 M LiPF_6_ in EC and EMC, 3:7 by weight) demonstrated decomposition beyond 4.9 V, highlighting the challenges in achieving stable high-voltage operation for Li-ion batteries. Fluorinated molecules exhibit increased oxidation potentials attributed to the strong electron-withdrawing influence of the fluorine atom [68]. In their study, Zhang et al. (2013) [69] explored the resilience of various fluorinated electrolytes under high-voltage conditions. Their findings revealed that E5 electrolytes (consisting of 1.2 M LiPF_6_ in fluorinated cyclic carbonate (F-AEC), fluorinated linear carbonate (F-EMC), fluorinated ethyl propyl ether (F-EPE) in a ratio of 2:6:2) demonstrated superior electrochemical stability compared to the EC/EMC-based electrolyte. Additionally, substituting EMC with F-EMC and EC with F-AEC notably enhanced the voltage tolerance of the electrolyte [68,69].

Lavi et al. (2020) [70] studied the impact of replacing traditional alkyl carbonate solvents with fluorinated cosolvents—fluoroethylene carbonate (FEC), difluoroethylene carbonate (DFEC), and 1,1,2,2-tetrafluoroethyl-2,2,3,3-tetrafluoropropyl ether (F-ETPE)—in high-voltage Li-ion cells charged up to 4.8 V, finding significant performance improvements. These enhancements were primarily due to stable, protective surface films formed on cathode particles, which isolated the cathode material from harmful reactions with the solvents. Testing with full graphite-Li_1.2_Mn_0.56_Co_0.08_Ni_0.16_O_2_ cells showed outstanding cycling performance over 1000 cycles using electrolyte solutions containing DFEC, F-ETPE, and 1 % tris(trimethylsilyl) phosphate (TMSP), indicating the potential for broader application in high-voltage cathode materials (Lavi et al., 2020) [70]. Fan et al. (2019) [71] introduced fluorinated electrolytes to overcome issues like high solvent-ion affinity and flammability associated with commercial non-aqueous Li-ion batteries, which are limited by operating temperature ranges of −20 to +50 °C and voltage ranges up to 4.3 V. These new electrolytes use combinations such as lithium bis(fluorosulfonyl)imide with fluoroethylene carbonate and 2,2,2-trifluoroethyl carbonate (LiFSI-FEC/FEMC), and lithium bis(pentafluoroethylsulfonyl)imide with fluoroethylene carbonate and diethyl carbonate (LiBETI-FEC/DEC), dissolved in highly fluorinated non-polar solvents like tetrafluoro-1-(2,2,2-trifluoroethoxy)ethane (D2) or methoxyperfluorobutane (M3). These electrolytes are non-flammable, offer high electrochemical stability across an expanded voltage window of 0.0–5.6 V, and maintain ionic conductivities across a wide temperature range from −125 to +70 °C. Testing showed that under these conditions, lithium-nickel-cobalt-aluminum oxide ((LiNi_0.8_Co_0.15_Al_0.05_O_2_) cathodes and aggressive lithium anodes, exhibited coulombic efficiencies exceeding 99 %, highlighting their potential for improved performance and safety in a broader range of operating conditions [71].

Kerner et al. (2016) [72] introduced new electrolyte compositions featuring thermally stable salts such as lithium 4,5-dicyano-1,2,3-triazolate (LiDCTA) and lithium 4,5-dicyano-2-trifluoromethylimidazolide (LiTDI), in solvent mixtures with high-flashpoint adiponitrile (ADN). When combined with sulfolane (SL) and EC as co-solvents, these electrolytes exhibit extended liquid temperature ranges without compromising flashpoint, albeit with higher viscosities and moderate ionic conductivities. Their anodic stabilities are suitable for lithium-iron phosphate (LFP) cathodes, maintaining coulombic efficiencies exceeding 99 % over 20 cycles in Li/LFP cells. Electrolytes with an ADN:SL solvent combination show excellent thermal stability at temperatures above 60 °C [72].

The replacement of liquid electrolytes with solid-state electrolytes (SSEs), including inorganic ceramic electrolytes and solid polymer electrolytes, has garnered increasing attention due to their notable advantages. These include enhanced safety, expanded electrochemical windows, a broad operating temperature range, and heightened thermal stability [73,74]. SSEs boast an electrochemical window that can extend up to 6V compared to metallic lithium, enabling the utilization of high-voltage cathode materials in Li-ion batteries. Additionally, SSEs could facilitate the adoption of lithium metal as an anode, offering an exceptionally high theoretical specific capacity (3860 mA h g^−1^), a low electrochemical potential (−3.04 V vs. the standard hydrogen electrode [SHE]), and a low density (0.53 g cm^−3^ at room temperature) [75].

In their study, Kautz et al. (2023) [76] underscored the pivotal role of electrolytes in determining the fast-charging capabilities of batteries by influencing ion transport and the resulting electrode/electrolyte interphases. Through their investigation of the impact of salt content, coordinating solvent, and non-coordinating diluent on the degree of salt dissociation and electrolyte ionic conductivity, they developed a controlled solvation structure electrolyte. This advancement enhances lithium ion mobility and conductivity while improving the kinetics and stability of electrode/electrolyte interphases, thus facilitating rapid charging of high-energy-density Li-ion batteries at rates of up to 5C (12-min charging) [76]. Logan and Dahn (2020) [77] discuss the ambitious target of achieving 'Extreme Fast Charging' (XFC)—charging batteries to 80 % capacity in less than 15 min. They highlight recent advancements in electrolyte formulations tailored for high-rate charging scenarios, including the use of low-viscosity co-solvents, highly concentrated solutions, and large polyanionic structures. Additionally, they explore innovative techniques for assessing the transport properties of electrolytes, as well as the significance of incorporating additives into the electrolyte to enhance performance [77].

Building on these advancements in electrolyte technology, Zhao et al. (2023) [78] explore further enhancements through the strategic use of additives in single-crystal LiNi_0.83_Co_0.11_Mn_0.06_O_2_ (SC-NCM83) cathodes. Their findings indicate that incorporating 1 % lithium difluoroxalate borate (LiDFOB) and 1 % lithium difluorophosphate (LiPO_2_F_2_) additives significantly enhances cycling performance, with a capacity retention of 93.6 % after 150 cycles, surpassing individual additive or baseline electrolyte performance. The combined additives promote the formation of a conformal cathode/electrolyte interface (CEI) layer, stabilizing the bulk structure and reducing impedance during long-term cycling. This enhancement is attributed to improved rate capability and cycling stability [78].

Zhou et al. (2024) [79] examined the latest developments in which ionic liquids function as electrolytes, dielectric layers, and structural components within single-molecule junctions, thereby transforming charge transport, redox reactions, and molecular dynamics in nanoscale environments.

Considering these advancements, it is imperative for future research to explore a broader range of materials, including non-traditional salts and solvents, which could offer additional electrochemical properties or environmental benefits. Researchers should also focus more on the compatibility between electrolytes and other battery components to maximize longevity and safety. This involves a deeper investigation into the dynamics of the electrode/electrolyte interphase to control unwanted reactions and degradation processes better. While laboratory-scale experiments provide foundational insights, scaling these findings to pilot and industrial levels is vital to assess their real-world application, durability, and economic feasibility. This transition from bench to market necessitates robust testing methodologies and partnerships with industry stakeholders. Furthermore, the adoption of sophisticated analytical techniques and predictive modelling can accelerate the development of novel electrolytes by offering deeper insights into molecular-level interactions and stability mechanisms under various operating conditions.

## 4 Solid electrolyte interface (SEI) (atomic level chemistries)

**Fig. 4:**
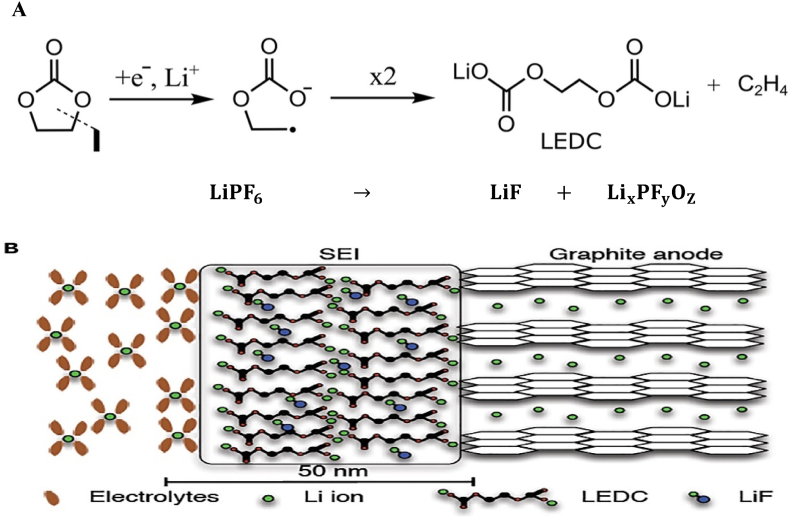
Schematic of the lithium plating formation process (discussed in later stages) as well as initial SEI Formation Mechanism and Composition (**A**) Initial reduction reactions of ethylene carbonate (EC) on the graphite electrode interface [83], (**B**) Illustration of the initial SEI formed on graphite surface during the first cycle of a Li-ion battery [84].

One impediment to improving EV battery performance is the electrode/electrolyte interface, which is critical to understanding battery electrochemistry because it is where the combination of electrons and metal ions takes place, followed by their storage in the electrode. This storage can occur through intercalation, alloying, or simply as a metal [80,81]. For example, a layer of lithium compounds can form on the anode electrode's surface when it interacts with the electrolyte solvent and salt, resulting from side reactions during the initial charging cycles (see Fig. 4A, which shows the reduction of the commonly used electrolyte solvent ethylene carbonate (EC) in Li-ion batteries, forming a layer on the negative electrode when reduced at potentials below 0.8 V vs. Li^+^/Li) [[82], [83], [84]]. This layer is commonly referred to as the solid electrolyte interface (SEI). The layer, composed of the reduced insoluble and partially soluble electrolyte components, forms instantly when the metal comes into contact with the solution. The thickness of the newly created layer is controlled by the electron-tunneling range [82,85,86]. Serving as an interphase region between the metal and the solution, this layer exhibits characteristics similar to those of a solid electrolyte, showing a high level of resistance to electron flow [87]. A passivation layer on the electrode, as discussed by Wang et al. (2018), usually complicates this solid electrolyte interface [80].

The SEI acts as a protective layer that prevents further reactions between the electrolyte and the anode and facilitates the transport of lithium ions through the anode [85]. Excessive SEI, however, can lead to capacity loss, shorter battery life, and safety hazards, while a stable SEI layer is necessary for the proper operation and performance of Li-ion batteries. The SEI layer serves several crucial functions in Li-ion batteries: i) Acting as a physical barrier: The SEI layer is an electrically insulating barrier that separates the electrode material from the electrolyte. This prevents the flow of electrons and ions, mitigating the risk of short circuits and other safety hazards. ii) Enabling ion transport: Despite being an insulator, the SEI layer facilitates the transport of lithium ions between the electrode material and the electrolyte, which is essential for the operation of Li-ion batteries. iii) Stabilizing the electrode material: The SEI layer contributes to stabilizing the electrode material by preventing further reactions between the electrode and the electrolyte. This minimizes degradation over time, prolonging the battery life [[88], [89], [90], [91], [92]]. Additionally, the SEI can function as an effective passivating layer, preventing further electrolyte decomposition and electrode exfoliation (see Fig. 4**B**) [84].

The formation and quality of the SEI layer can impact not only the charging and discharging rates of an EV battery but also its capacity fade [93]. A thick and high-quality SEI layer can limit the flow of ions in and out of the battery during charging, resulting in slower charging speeds. Conversely, a thin and low-quality SEI layer can allow for faster charging, but it may also lead to decreased battery efficiency (reduced battery performance) and a shorter overall lifespan [94]. As the battery is discharged, the SEI layer can degrade over time, resulting in reduced battery capacity and shorter run times. A high-quality SEI layer can help to maintain a more stable discharge rate, while a low-quality SEI layer may lead to more rapid discharge and reduced battery performance [95,96]. Thus, it is widely acknowledged that the SEI layer is critical to the performance of Li-ion batteries, influencing initial capacity loss, self-discharge characteristics, power capability, the morphology of lithium deposits, cycle life, shelf life, rate capability, and safety [87].

While the existence of the anode SEI layer is critical, controlling its formation (seeding) and growth is challenging due to the impact of various factors on its morphology, chemical composition, and stability. These factors include cell temperature, graphite type, electrolyte composition, electrochemical conditions, and morphology. To prevent concentration polarization and aid in the deposition and subsequent dissolution processes of the metallic anode, the cation transport number must be near unity. The SEI should exhibit excellent flexibility, mechanical stability, and strong adhesion to the anode, which are critical parameters [97]. Therefore, prioritizing the formation of the SEI layer and ensuring its electrochemical stability is imperative for prolonged operation in the development of future Li-ion battery technology [98].

In order to minimize the amount of SEI formation in Li-ion batteries, a combination of approaches is considered: i) Using high-quality electrolytes with low impurities and high stability. These electrolytes should contain at least one solid-electrolyte interphase (SEI) precursor that reacts rapidly with an alkali-metal anode or lithium, producing an insoluble SEI. ii) Adding SEI-forming additives to the electrolyte can also help create a stable SEI layer that prevents further reaction with the electrolyte. Common SEI-forming additives include fluoroethylene carbonate (FEC), lithium difluoro(oxalato)borate (LiDFOB), vinylene carbonate (VC), and lithium bis(oxalate)borate (LiBOB). iii) Following proper charging and discharging protocols is important to avoid charging or discharging the battery too quickly or slowly, as well as overcharging or over-discharging the battery. iv) Reducing the battery temperature can also help minimize the formation of SEI, as high temperatures can accelerate its formation. v) Optimizing the electrode materials by considering their composition and structure can also be beneficial [99,100]. Researchers, including Beheshti et al. (2022), An et al. (2016), and Meng et al. (2022), have been exploring new electrode materials to form a more stable SEI and reduce the formation of excessive SEI. vi) Implementing active SEI layer management is a new and promising approach that involves creating an active layer that can continuously manage and repair the SEI. It involves adding a catalyst to the SEI layer that can promote the formation of a more stable SEI and thus repair any damage to the layer [86,87,101].

Researchers, including Lin, Liu, and Cui (2020); Manthiram (2019); Zhang et al. (2021); Li, Han, and Liu (2020); Aurbach, Lu, and Schechter (2016); and Cheng and Zhang (2014), have provided a comprehensive overview of SEI formation mechanisms and mitigation strategies [87,89,[102], [103], [104], [105]]. Despite these advancements, further refinements are necessary to fully harness the benefits of SEI for enhancing battery performance and longevity.

While the contributions of the research are commendable, several areas require additional exploration to deepen our understanding of SEI dynamics and to translate these findings into practical solutions. Continued research into the molecular and atomic interactions within the SEI layer, using advanced spectroscopy and microscopy techniques, could reveal the precise mechanisms at play and enable the design of more effective SEI layers. Exploring new materials for the anode, electrolyte, and SEI additives can open up avenues for batteries with higher efficiency and safety profiles. For instance, the use of novel nanostructured materials might offer improved ion transport and stability. Additionally, research focusing on greener synthesis methods and recycling processes for battery components, particularly those involved in SEI formation, would be beneficial and have minimal environmental impact. Transitioning from laboratory-scale research to real-world applications often presents unforeseen challenges; therefore, extended field testing under varied environmental conditions can help ensure the robustness of SEI innovations. Finally, developing more sophisticated computational models to predict SEI behaviour under different conditions can significantly speed up the innovation cycle. These models allow for the simulation of long-term performance and the identification of potential improvements without the need for extensive physical testing.

## 5 Battery separators and improvements

Further, battery separators are key components that provide a barrier between the anode (negative) and the cathode (positive), enabling the smooth flow of ions from one side to the other and thus preventing short-circuiting within the cell. The separator's functionality impacts safety, longevity (i.e., durability), and battery performance [106,107]. According to Zhang et al. (2021), a highly safe separator must possess the following characteristics: (i) Exceptional heat resistance that prevents significant shrinkage at high temperatures and may even possess flame retardant properties; (ii) Exceptional properties that prevent dendritic growth; (iii) Strong mechanical properties to maintain the separator's structural integrity, whether it is constructed as an independent component or integrated directly into the battery device; and, (iv) Excellent chemical compatibility to prevent reactions from taking place between the separator and the rest of the battery's parts and thus exhibit a strong affinity for the electrolyte [106]. Research on separators aims to improve properties such as thermal stability, mechanical strength, electrolyte retention, and ion conductivity [108,109].

Therefore, developing a high-safety separator that meets these performance requirements is crucial and should be a top priority in battery development. Researchers, including Zhang et al. (2021) and Liu et al. (2020), have directed their attention toward high-safety separators, involving modification of existing polyolefin (polypropylene-PP, polyethylene-PE) separators and the development of new separators using novel materials and structures. Composite separators are produced by leveraging the mechanical strength and chemical stability of commercially available PE and PP separators, achieved through the application of a coating or incorporation of organic or inorganic materials onto the surfaces of these separators. As a result, these composite separators exhibit excellent properties [106,110].

Leng et al. (2022) and Xing et al. (2022) have suggested the use of electrospinning technology to fabricate nanoporous structured nanofiber (electrospun) separators. These separators showcase notable characteristics, including high porosity, robust physical strength, minimal thickness, a large specific surface area, and superior wetting properties. However, it's important to note that the mechanical strength of electrospun separators is relatively low. Consequently, significant tension is exerted during the battery assembly process, which is considered unfavourable [107,111]. Research is essential for exploring advanced fibre materials and post-treatment processes that enhance membrane mechanical strength—a necessity for ensuring the commercial viability of electrospun battery separators. Additionally, there is consideration for enhancing electrospun separators with controllable nanostructures, such as core-shell, hollow, multi-channel, etc. This potential enhancement could introduce innovative approaches to designing and fabricating high-performance separators. Both theoretical and experimental studies are vital for understanding and improving the heat transfer properties within electrospun separators [107,112].

Recent developments in EV battery separator technology include using advanced materials such as graphene, ceramic, and polymers. Graphene oxide and its derivatives have shown promise as separator materials due to their excellent mechanical, thermal, and electrical properties [112]. Wang et al. (2019) invented a heat-resistant battery with good electrolytic wettability, using polyimide (PI) as a primary material, incorporating graphene oxide (GO) as a reinforcing agent, and employing electrospinning technology to develop the nanofiber membrane. The resulting structure demonstrated no shrinkage even up to 200 °C, exhibiting excellent thermal stability, high electrolyte absorption rates, improved ionic conductivity (>2.0 mS/cm), excellent cycling performance, and better C-rate discharge capability [113]. Researchers such as Roh et al. (2022) and Kang et al. (2022) report ongoing exploration of ceramic and polymer composite materials to enhance the separator's ion conductivity and thermal stability. These materials effectively suppress the separator's thermal shrinkage even in unusual situations, leading to a safer battery [114,115]. Moreover, efforts have been made to improve the manufacturing process for producing high-quality separators. This involves the development of scalable production methods, such as electrospinning, phase inversion, and dry-casting techniques. The focus is on methods that can produce high-performance separators with consistent quality and at a lower cost than conventional methods [116,117].

While recent studies have primarily concentrated on enhancing the heat resistance, chemical compatibility, and structural integrity of separators, there remains a significant need to strengthen their mechanical properties, particularly for separators developed using innovative materials such as electrospun nanofibers. Research has highlighted the potential of nanoporous structured nanofibers but also pointed out their low mechanical strength under tension during battery assembly. Addressing these challenges by developing advanced fibre materials and post-treatment processes is crucial for ensuring the commercial viability of these innovative separators. Furthermore, ongoing research into materials like graphene oxide and ceramic composites should continue, as they show significant improvements in thermal stability and ionic conductivity, potentially leading to breakthroughs in separator performance. Innovative nanostructures should be further explored to achieve high functionality and scalable production despite existing challenges in this area. It is essential to refine and enhance manufacturing techniques such as electrospinning, phase inversion, and dry-casting to ensure the production of high-quality separators at a lower cost, facilitating the practical widespread use of these innovations.

## 6 Cathode chemistries and improvements

Improvements have been made to both the composition and structure of the electrode (cathode and anode) materials, as they play a crucial role in determining the mobility of metal ions in the battery cell. For example, research has focused on the composition and materials used in manufacturing the cathode, yielding various types of cathodes such as lithium-manganese oxide (LMO), lithium-iron phosphate (LFP), lithium-nickel-manganese-cobalt oxide (NMC), and lithium-nickel-cobalt-aluminum oxide (NCA). This clearly shows that the cathode is composed of different lithium compounds, imparting distinct characteristics to each battery, such as capacity, power density, cycle life, and safety. Nitta et al. (2015) demonstrated that LFP batteries have a long cycle life, a high power density, good thermal stability at high temperatures, and superior electrochemical performance [118]. On the other hand, Shu et al. (2021) argued that EV batteries with lithium-cobalt oxide (LCO) cathodes have high specific energy but low specific power, making them less suitable for high-load applications but exhibiting longer durations in power delivery [119]. Mahmud et al. (2022) have shown that LCO has a higher mobility of lithium ions when compared to other cathode materials, such as LFP [120]. Notably, the cathode material in LMO batteries enables the production of a three-dimensional structure that enhances thermal stability and safety while also improving ion flow, decreasing internal resistance, and increasing current handling (current carrying capacity) [121]. Sun et al. (2015) [122] presented a novel method to construct LiMn_2_O_4_ (LMO) nanowires coated with amorphous carbon, circumventing the need for templates. The challenges associated with achieving one-dimensional growth, stemming from the crystal structure of LMO, were successfully overcome. The resulting electrode demonstrates exceptional rate performance and high capacity for extended cycling, maintaining 82 % of its initial capacity even after 1500 cycles at a high current density. Their research findings further suggested that the application of this innovative strategy extends beyond the confines of LMO nanowires. It enables the successful synthesis of other carbon-coated manganese-based nanowires. This approach holds promise for the development of ultrafast rechargeable Li-ion batteries and other advanced one-dimensional materials, leading to enhanced electrochemical performance in energy storage devices [122].

The benefits of the three main cathode elements are combined in NMC batteries: nickel, manganese, and cobalt. Nickel possesses high specific energy, providing greater storage capacity. It delivers greater energy density at a lower cost, but it lacks stability on its own [123,124]. Manganese exhibits outstanding rate capability and enhances the battery's safety by enabling high current discharge while maintaining low temperatures, ensuring extreme stability and satisfactory cycle durability despite its low specific energy [125,126]. On the other hand, cobalt is commonly used in most cathodes of Li-ion batteries, stabilizing the layered structure and offering high energy density [127]. When combined, these elements produce a high-specific-energy, stable chemistry. Employing a battery system based on NMC chemistry allows for the utilization of the advantageous properties of all three elements: manganese, nickel, and cobalt. This configuration offers several benefits, such as a relatively high capacity, the ability to handle high loading capacity, and cost savings, as the battery does not necessitate complex built-in circuits [[128], [129], [130]]. It is worth noting that lithium-layered NMC is the most commonly preferred option for cathode material. However, it faces challenges such as cation mixing, changes in volume, microcracking, surface-related side reactions, high-temperature performance issues, cycling stability, and structural reconstruction [131,132]. Many researchers, Chen et al. (2021), Luo et al. (2021), and Song et al. (2020), have conducted research on the development of NMC electrodes with the aim of addressing the challenges as mentioned above, increasing energy density, and optimizing schemes [[132], [133], [134]]. Sun et al. (2018a), Sun et al. (2018b), and Fu et al. (2023) highlight a significant shift towards cobalt-free cathode materials in the Li-ion battery industry, driven by the need to reduce production costs and environmental impact [[135], [136], [137]]. Sun et al., 2018a, propose the development of a hierarchical porous micro/nanostructure that is crucial for enhancing the performance of the lithium-nickel-manganese oxide (LiNi_0.5_Mn_1.5_O_4_ (NMO)) cathode. This was achieved by employing an efficient solvothermal method to construct waxberry-like NMO with interconnected single-crystalline nanoparticles, using ethylene glycol and hexamethylenetetramine as a precipitant. The result was a uniform micro/nano structure with lower impurity levels. The resulting material exhibited excellent rate capability and maintained about 84 % of its initial capacity after 1200 cycles at a high discharge rate of 30 C, suggesting promising implications for advancing high-performance energy storage devices [135]. Sun et al. (2018b) conducted a study to address the challenge of porosity and crystal orientation in cathode materials. Their innovation led to the synthesis of hierarchically porous urchin-like NMO hollow spheres featuring prominently exposed {111} facets, utilizing MnO_2_ nanosheets and polystyrene spheres as precursors. They emphasized the unique crystal orientation, which reduces manganese dissolution and ensures impressive rate capability and excellent cyclic stability. The cathode electrode material retained 92 % of the initial discharge capacity after 1500 cycles, showcasing the high potential for use in high-energy-density Li-ion batteries [136]. Fu et al. (2023) focused on the high-voltage spinel NMO, standing out for its increased voltage platform, theoretical energy density, and cost-effectiveness. However, its limited cycle performance and vulnerability to high temperatures have impeded widespread adoption. They propose exploring future research avenues, including the integration of machine learning algorithms in materials design and AI-assisted virtual experiments, to enhance electrochemical performance [137]. Current nickel-rich and cobalt-free cathode materials are still in the experimental stage, with certain technical problems remaining challenging. These issues encompass concerns ranging from material synthesis and processing to the overall performance, safety, and durability of batteries utilizing these cathodes [138]. Researchers are actively addressing these challenges, employing innovative methods such as material engineering—specifically, the application of machine learning algorithms in materials design—surface coating techniques, and novel approaches, like leveraging computational models to predict and optimize performance. These efforts aim to enhance the overall performance and stability of cathode materials [139].

Lithium titanate oxide (LTO) batteries use either LMO or NMC as the cathode chemistry, and graphite in the anode is replaced with lithium titanate. As a result, the battery is extremely safe, has a long lifespan, and charges more quickly than any other lithium battery system. Newer systems combine these chemistries at different ratios to improve the battery properties. For example, combining LCO, LMO, nickel, aluminium, iron, and phosphate enhances longevity, loading capabilities, and safety while also reducing costs [128,129,140].

NCA batteries provide a notable combination of high specific energy, adequate specific power, and a prolonged lifespan. The inclusion of aluminium in these batteries contributes to their stability, substantial capacity, and extended cycle life. Consequently, NCA batteries can deliver a relatively high current level over prolonged durations. The cathode material's chemical composition differs from the cathodes of the previous lithium battery types discussed [141]. Ongoing research aims to enhance the energy density of NCA batteries, crucial for applications demanding longer driving ranges in electric vehicles or greater energy storage capacities, with a specific focus on exploring new electrode materials, optimizing electrode structures, and improving overall battery design without compromising other performance metrics [142].

To further improve cathode materials, research efforts should prioritize addressing key challenges identified in existing cathode chemistries, including enhancing intercalation efficiency, stability, and rate capability, while ensuring safety and durability. Studies have shown promising advancements in cathode materials such as LMO, NCA, and NMC, which offer high energy density and stability. However, researchers focusing on NMC and NCA chemistries have noted significant issues like cation mixing, volume changes, and microcracking that can degrade battery life. Future research should focus on optimizing cathode structures and enhancing structural stability through advanced material engineering techniques, such as atomic layer deposition or advanced doping strategies. Given the ethical and environmental concerns associated with cobalt mining, continued efforts are needed to develop efficient cobalt-free cathodes. Further improvements should be pursued by utilizing machine learning and computational modelling to expedite the discovery of new materials that can match or exceed the performance of cobalt-containing cathodes. While materials like LMO and LTO offer enhanced safety features, there is always room for improvement, especially under extreme conditions. Research into novel electrolyte formulations or solid-state battery technologies could provide breakthroughs in safety without compromising battery performance. As highlighted by various studies, the cost of materials and manufacturing processes remains a significant barrier to the widespread adoption of advanced Li-ion batteries. Therefore, research into more cost-effective synthesis methods, recycling of battery components, or alternative, cheaper materials that do not compromise on performance would be beneficial.

### 6.1 Selected high-quality cathode electrodes for improved performance

Advancements in Li-ion battery technology significantly depend on the development of superior cathode materials, which play a crucial role in enhancing overall battery performance, energy density, and stability. Among the diverse range of cathode materials employed, LCO, NMC, and LFP stand out due to their distinct properties and applications. These high-quality electrode materials, discussed herein, can significantly improve the performance of Li-ion batteries.

LCO is a widely used cathode material known for its high energy density and stability. High-purity LCO is particularly dominant in portable electronic applications, boasting a commercial capacity of approximately 140 mA h g^−1^ and a theoretical capacity of 274 mA h g^−1^ [143].

NMC cathodes provide a balance of high energy density, power capability, and cycling stability. High-quality NMC materials with precise stoichiometry and low impurity levels enhance the overall performance and lifespan of Li-ion batteries. Conversely, Ni-rich NMC cathode materials offer higher energy density due to their high nickel content. However, improper synthesis techniques can result in structural defects, phase impurities, or uneven distribution of elements. These factors have the potential to decrease the battery's overall capacity and energy density [144]. A study conducted by Zhang et al. (2020) [145] investigated the impact of Cu impurities in different forms within the NMC622 cathode. They found that Cu metal impurities negatively affect the electrochemical performance of the NMC622 cathode, while copper ion impurities that replace Ni^2+^ sites reduce cation mixing within the NMC622 cathode structure. The NMC622 cathode containing 0.34 % Cu ion impurities showed optimal performance, achieving higher initial charge and discharge capacities of 211.8 and 186.0 mA h g^−1^ at 0.05C, respectively. These values exceed those of the virgin NMC622 cathode by 12.2 and 14.1 mA h g^−1^ [145]. The findings strongly suggest that the presence of Cu metal impurities should be avoided and that Cu ionic impurities should be carefully managed at optimal levels [145,146].

High-quality LFP (LiFePO_4_) electrodes with minimal impurities contribute to improved battery performance, especially in applications that prioritize safety, stability, and durability. Although LFP naturally occurs as the mineral triphylite, its impurities can detrimentally affect its electrochemical properties, rendering triphylite inefficient for use. The synthesis process for LFP is crucial as it determines the microstructure of the compound, which profoundly influences performance [147].

## 7 Anode chemistries and improvements

The other part of the electrode is the anode which offers the most challenging issues in battery cell design, such as achieving high capacities and ensuring functional optimality at high operating rates. Due to Pigłowska et al. (2021), research has dealt with the development of fast-charging anode materials [148]. Other researchers, namely Nzereogu et al. (2022) and Deng, and He, (2023), conducted similar studies. They utilized various anode components, including (i) carbon-based alternatives such as carbon nanotubes and 'graphite materials' (i.e., graphene or graphene oxides); (ii) metal-based composites like lithium-titanium oxide (LTO), titanium oxide (TiO_2_), LMO, cobalt tetraoxide (Co_3_O_4_), iron oxide (Fe_3_O_4_), and nickel oxide; and (iii) alloy composites like silicon and tin-based compounds. These materials demonstrated notable levels of success [149,150]. Their results verified the fact that conventional graphitic anodes are characterized by potentials that are relatively close to the Li/Li^+^ formation potential. This makes them useful for maximizing cell energy density but leaves them vulnerable to Li plating. Most EV batteries available on the market are made out of lithium, so lithium is used to test for their performance. In this vein, Chen (2013) has indicated that one of the most successful strategies for enhancing the Li-ion cells' ability to charge quickly has been the modification of the anode materials. This is achieved by coating them with other materials [151]. For example, Kim et al. (2019) have noted that surface-engineered graphite with a coating of merely 1 wt% aluminium oxide (Al_2_O_3_) displays a reversible capacity of approximately 337.1 mA h g^−1^ at moderate rates. At high rates, it reaches 4000 mA g^−1^ [152]. Further, Dong et al. (2020) and Wu et al. (2021) have noted that applications for high-power Li-ion batteries could make use of specific metal oxide and alloy-based materials, but due to their inherent extreme volume change, pulverization and primary particle agglomeration, and weak electronic conductivities, these materials are frequently constrained in terms of their fast charge useable capacity and cycling stability [153,154].

Some researchers have utilized nanotechnology to enhance EV battery performance, including studies by Mohan et al. (2018) [155], Khan, Saeed, & Khan (2019) [156], Wu et al. (2011) [157], Yang et al. (2016) [158], Yu et al. (2022) [159], Anasori & Gogotsi (2019) [160], Li et al. (2016) [161], Yun et al. (2022) [162], Ding et al. (2016) [163], Perera et al. (2023) [164], Boaretto et al. (2021) [165], Li et al. (2022) [166], Guo & Han (2022) [167], Liu et al. (2018) [168], Qian et al. (2015) [169], and Griffith et al. (2018) [170]. These studies [[155], [156], [157], [158], [159], [160], [161], [162], [163], [164], [165], [166], [167], [168], [169], [170]] investigated the use of nanomaterials to improve the anode electrode. The progress made in nanocomposites based on graphene and polymers has emerged as one of the most significant additions. Graphene is a two-dimensional structure consisting of carbon atoms arranged and organized in a hexagonal crystalline structure with sp2 bonds. It possesses several remarkable properties, such as a high theoretical specific surface area (making it highly effective for adsorption and surface reactions), offers high intrinsic carrier mobility with impressive thermal and electrical conductivity, and exhibits improved mechanical strength [155]. Because of its exceptionally large surface area compared to other similar materials, it facilitates better interaction between the graphene sheets and the polymer material. Consequently, it finds applications in a variety of fields, including the biomedical and electronics industries [156]. Graphene-based materials can be used as anode materials by either integrating with Si and Sn, which have a substantial volume change or by N-doping to provide a high reversible capacity exceeding 1040 mA h g^−1^ at a current density of 50 mA g^−1^ [157]. Yang et al. (2016), in a study they conducted, described a high-performance anode electrode for Li-ion batteries. These researchers developed a binder-free carbon-coated silicon/reduced graphene oxide (Si/rGO) nanocomposite using electrophoretic deposition. This anode demonstrated remarkable characteristics, notably a higher reversible specific capacity of 1165 mA h g^−1^ at 0.1 Ag^-1^, which is three times greater than that of graphite. It exhibited outstanding cycling stability, retaining approximately 96.8 % capacity at 1 A g^−1^ and around 95.4 % capacity at 2 A g^−1^ after 100 cycles [158]. 2D potential anode materials that are similar to graphene enable faster electron transport, more increased ion storage sites, and reduced Li^+^ diffusion pathways because of their high surface-to-mass ratio and distinctive physicochemical properties [159]. These 2D anode materials are primarily made of transition metal carbides/nitrides components (primarily highly conductive MXenes, i.e., titanium carbides (Ti_2_CT_x,_ Ti_3_C_2_T_x_, Mo_2_Ti_2_C_2_T_x_, Cr_2_TiC_x_T_x_, Nb_4_C_3_T_x_, and V_2_CT_x_), transition metal dichalcogenides (TMDs, i.e., MoS_2_, MoSe_2_, WS_2_, WSe_2_, FeS, FeS_2_, and CoS_2_), as well as transition metal oxides (TMOs, i.e., NiO, Co_3_O_4_, MnO_2_, and Fe_3_O_4_), and oxides as potential anode materials [150,160]. In a recent development, the Goodenough group proposed a titanium niobium oxide (TiNb_2_O_7_) anode with a high-rate capability that rivals the theoretical capacity of graphite. This anode demonstrates rapid intercalation and or deintercalation of Li^+^ ions with an extended lifespan, indicating its potential as a viable alternative to LTO (Li_4_Ti_5_O_12_) anodes [161].

The integration of two-dimensional materials into three-dimensional macroscopic structures like porous films, scaffolds, and networks can intensify the transportation of ions and electricity within electrode materials [162]. Utilizing 3D hierarchical structures, such as molybdenum disulphide (MoS_2_) with column-like structures, can achieve a high reversible specific capacity of 840 mA h g^−1^ at 200 m A h g^−1^, outstanding rate performance, and cyclic stability of up to 500 cycles [163]. Since its discovery in 2011, the electrically conductive and electroactive MXenes have attracted significant interest. Strategies such as the fabrication of two-dimensional MXene flakes into hollow spheres, the construction of three-dimensional structures, and the development of thick, vertically aligned MXenes with high-rate charging/discharging capabilities have been successfully proposed to achieve three-dimensional hierarchical architectures [164].

Metallic Li is widely favoured as an anode material for its ability to enhance energy density; however, due to the pure Li foil's limited surface area, it suffers from low power density. Nevertheless, the rate capability can be improved by incorporating lithium metal into 3D structural matrices, facilitating faster diffusion of Li-ions. For instance, a composite material consisting of a 3D porous electrospun carbon matrix infused with lithium melt exhibited stable cycling and exhibited low overpotential (below 90 mV) during Li plating or stripping [149,165].

The selection of anode materials, their modification, and nanoscale structure design are not the only methods for improving the fast-charging capability. The performance of anodes is also significantly influenced by electrolytes and interfaces, impacting factors such as ion conductivity, charge transfer kinetics, and the formation of the solid-electrolyte interface (SEI) layer [166,167]. Alternative methods for optimizing the interface between the anode and electrolyte involve making improvements to the way amorphous carbon coatings are applied to graphite. These coatings aid in creating a more consistent SEI layer, which helps suppress the formation of undesirable (problematic) active sites on the graphite surface where lithium deposition is prone to occur. Optimizing the anode/electrolyte interface can be achieved by applying metal coatings and introducing dopants such as copper (Cu) and tin (Sn) to the anode. Moreover, selecting the appropriate lithium salt and co-solvents is crucial in achieving an optimized interface [168].

According to Qian et al. (2015), lithium metal anodes with a high rate of non-dendritic (dendrite-free plating of Li metal at high rates) and high Coulombic efficiency, employing extremely concentrated electrolytes have been developed. A high Coulombic efficiency of 98.4 % was attained when a copper/lithium (Cu/Li) cell was subjected to 41,000 cycles at a current density of 4 mA cm^−2^, using a lithium Bis(fluorosulfonyl)imide–1,2-dimethoxyethane (LiFSI/DME) electrolyte with a concentration of 4 M [169]. Griffith et al. (2018) reported niobium tungsten oxide (Nb_16_W_5_O_55_ and Nb_18_W_16_O_93_) anode materials that showed outstanding high-rate performance. This was achieved without the use of nanoscale technology, providing a potentially affordable method to develop fast-charging electrode materials. The electrode microstructure has also been optimized in order to promote faster diffusion and increase rate capability [170].

Zhang et al. (2019) conducted a study on the influence of different preparation conditions on the alignment of graphite flakes by utilizing a magnetic field. They discovered that a higher degree of alignment resulted in shorter transmission pathways for Li^+^, which facilitated better diffusion of Li^+^ along these paths. As a result, Li-ion batteries exhibited improved rate performance. They observed that vertically aligned electrodes with a loading of 8.9 mg cm^−2^ achieved a specific capacity of 59.1 mAh g^−1^ at 2C, which was 4.5 times greater when compared to the reference electrode [171]. Li et al. (2019) reported the fabrication of LCO electrodes and meso-carbon microbead graphite. They utilized a low magnetic field and exhibited ultrahigh areal capacity (∼14 mAh cm^−2^ vs. 2 to 4 mAh cm^−2^ for conventional lithium-ion) with directional pore arrays. These electrodes have been shown to deliver larger areal capacities sustainably (>10 mAh cm^−2^) than standard LCO electrodes at high C-rates [172]. Lithium diffusion is also influenced by crystal structure in conventional carbon or graphite anodes, affecting the overall performance. Fang et al. (2015) examined the electrochemical abilities of anodes made of mesophase soft carbon (MSC), mesophase graphite (SMG), and hard carbon (HC). Their findings revealed that MSC displayed the highest specific capacity at higher C-rates, which can be attributed to its larger interlayer spacing. Due to its higher crystallinity, SMG demonstrated a lower capacity at high C-rates when compared to the other two materials. This can be attributed to its smaller interlayer spacing, which hindered the diffusion process negatively [173]. Despite the fact that studies and research on Li-ion battery anodes fabricated from carbon-based materials have reached significant heights and nearly achieved perfection, there remains an opportunity for conducting extensive research on graphite materials to enhance magnification and current capacity. Future studies will prioritize the dynamic behaviour of carbon anode materials, considering economic (price) and safety factors [149].

Many studies have shown that the decay of the positive electrode and the growth of the positive cathode electrolyte interface (CEI) film does not influence the fast-charging speed of the traditional Li-ion system. Consequently, the negative electrode has become the primary focus during the charging process [150,[174], [175], [176]]. The practical implementation of lithium metal anodes in rechargeable Li-ion batteries encounters several significant challenges: i) Under specific conditions, lithium metal may continue to precipitate into dendrites or penetrate through the diaphragm, making contact with the cathode. This scenario can lead to internal short circuits and potentially catastrophic issues [150]. ii) The metallic lithium's high reactivity leads to the formation of a SEI, which negatively impacts the Coulombic efficiency of batteries and induces irreversible and ongoing interactions between lithium metal and electrolyte. iii) Unlike intercalated anodes like silicon and graphite anodes, lithium metal anode's virtually infinite volumetric change during repeated plating and stripping processes can cause severe damage to the electrode structure. iv) Lithium dendrites, when electrically isolated from the current collector and encapsulated by the insulating SEI, lose their electrochemical activity and are known as 'dead Li.' These 'dead Li' formations during repetitive plating and stripping cycles contribute to a substantial capacity degradation in Li-ion batteries. Adding to the challenge, these four issues mentioned above are interconnected rather than mutually independent, making the utilization of lithium anodes even more complicated. As a result, the practical implementation of lithium metal anodes in rechargeable batteries is still an ongoing challenge [175,177].

To improve the materials used for anodes in EV batteries, it is crucial to explore alternative components and enhance existing technologies for better energy storage and release. Key focus areas include the use of carbon-based materials, metal-based composites, and alloy composites, which have already shown success in increasing capacity and functionality at high rates. Additionally, modifying anode materials by coating them with carbonaceous materials, metal oxides, or polymers can help address issues such as susceptibility to lithium plating while preserving high energy density. The integration of nanotechnology, particularly graphene-based materials, has demonstrated significant improvements in anode performance, indicating the importance of further research to optimize reversible capacity and cycling stability. The exploration of 2D materials like transition metal carbides, nitrides, and oxides could also enhance electron transport and ion storage, potentially speeding up charging times. Recent studies on materials like TiNb_2_O_7_ suggest they are promising alternatives that could enable faster lithium intercalation and longer battery life. Challenges such as dendrite formation, SEI creation, volumetric changes, and capacity loss in lithium metal anodes continue to persist, necessitating further research for their effective use in rechargeable batteries. While these new materials and technologies show potential in laboratory settings, future research should also address their economic and practical scalability, cost-effectiveness, resource availability, and environmental impact. In summary, ongoing research into new anode components, material advancements, nanotechnology integration, and novel materials is essential for advancing anode technologies in EV batteries.

### 7.1 Selected high-quality anode electrodes for improved performance

High-quality graphite with low impurity levels is crucial for achieving optimal battery performance and cycle life in Li-ion battery anodes. This is due to its conductivity, cost-effectiveness, high reversible capacity, appropriate potential profile, and stability. Typically, mined graphite falls short of the necessary purity levels for EV battery-grade applications, necessitating additional purification steps involving chemical and thermal treatments to achieve the required purity, exceeding 99.5 %, for Li-ion battery anodes [178]. Among natural graphite types, vein graphite typically exhibits exceptional crystallinity and very high natural purity, reducing the need for extensive purification and thus cutting costs. The vein graphite variety studied by Hewathilake et al. (2017) [179] demonstrated exceptionally high purity and modified surface characteristics. Testing with galvanostatic charge-discharge, cyclic voltammetry, and impedance analysis showcased a stable reversible capacity of 378 mA h g^−1^, surpassing the theoretical capacity of LiC_6_ (372 mA h g^−1^). Additionally, the low irreversible capacity observed contributes to a high coulombic efficiency, exceeding 99.9 %. Hence, this highly crystalline vein graphite represents a cost-effective, readily deployable anode material for Li-ion rechargeable batteries [179].

Silicon has emerged as a promising candidate for high-capacity anode materials in Li-ion batteries. High-quality silicon-based anodes, typically in the form of silicon nanoparticles or composites, can significantly improve battery energy density and cycling stability [180].

When these materials are synthesized and utilized with attention to purity and microstructural control, they can greatly enhance the performance, efficiency, and longevity of Li-ion batteries.

## 8 Electrolyte-electrode side reactions (atomic level)

In ideal conditions, the cathode electrolyte interfacial (CEI) and solid electrolyte interphase (SEI) layers of a battery passivate the anode and cathode to prevent ongoing side reactions between the electrolyte and electrodes [85,181,182]. However, these protective layers aren't perfect and can contain regions where side reactions persist. These side reactions, occurring on both the anode and cathode surfaces, contribute significantly to battery degradation. When operating at small currents, the side reactions are controllable, resulting from reduced overpotentials and potentially lower operating temperatures, which allows commercial Li-ion batteries to achieve an acceptable cycle life. However, under high current densities, especially in extreme fast-charge (XFC) conditions, the local current distribution may become uneven. This can lead the battery system to experience significant Joule heating due to electron and ion transport/flow resistance within the battery system [2,[183], [184], [185]]. The temperature rise will enhance the speed of side (parasitic) reactions involving electrolyte components at each electrode. The overpotential that each electrode encounters will simultaneously shift the anode potentials towards more cathodic and the cathode potentials towards more anodic. From a thermodynamic perspective, this creates a stronger driving force for these undesired parasitic reactions to occur. These reactions could include species reduction at the anode, possible corrosion of the aluminium (Al) current collector, and oxidation of electrolyte species at the cathode, some of which could result in gaseous compounds as side reactions [186,187]. It is worth noting that there have been studies indicating insignificant temperature changes. These studies involved a ∼2 mAh cm^−2^ single-layer LiNi_0.5_Mn_0.3_Co_0.2_O_2_ with graphite anode pouch cell when charged at rates up to 9C [182,188]. A summary of reviews and discussions related to improvements in EV batteries is shown in Table 1.

**Table 1:** A summary of reviews and discussions related to improvements in EV batteries.

Category	Improvement	Parameter	Reference
Anode electrode	Use of Carbon-based (graphite, graphene, graphene oxides)	Maximized cell energy density, high lithium ion mobility, Susceptible to Li plating	Tomaszewka et al. (2019), Nzereogu et al. (2022). [2, 149]
	Metal composites, i.e., Titanium oxides (TiO_2_), Lithium-titanium oxides (LTO), LMO, Cobalt tetraoxide (Co_3_O_4_), Iron oxide (Fe_2_O_4_), Nickel oxide (NiO), Alloy Composites, i.e., Si and Sn.		
	Graphite + Al_2_O_3_	Reversible capacity	Kim et al. (2019). [152]
	Polymer nanocomposites based on graphite (graphene)	High intrinsic carrier mobility, outstanding thermal and electrical conductivity, and improved mechanical strength	Mohan et al. (2019). [155]
	Carbon-coated Si/rGO nanocomposite electrode + Li-ion battery	High reversible specific capacity, excellent cycling stability	Wu et al. (2011). [154]
	TiNb_2_O_7_ anodes	High theoretical capacity comparable to graphite, quick Li^+^ intercalation and deintercalation, longer lifecycle	Li et al. (2016). [161]
	MoS_2_ with 2D and 3D structures	High reversible specific capacity, outstanding rate performance, high cyclic stability	Perera et al. (2023). [164]
	Metalic Li	High energy density, low power density	Nzereogu et al. (2022), [149]
	Metalic Li + 3D structural matrices electrospun	Improved rate capability, faster Li-ion diffusion, stable cycling, low overpotential for Li plating or stripping	Nzereogu et al. (2022), Boaretto et al. (2021). [149, 165]
	Niobium tungsten oxides anode	High-rate performance, no nanoscalling, fast charging electrode materials, faster diffusion, and increased rate capability.	Griffith et al. (2018). [170]
	LCO + Meso-carbon microbial graphite anodes	Ultra-high areal capacity, High C-rates	Li et al. (2019). [172]
	MSC, SMG, HC	Good specific capacity at high C-rates	Fang et al. (2015) [173].
Cathode electrode	Use of LFP for the cathode	Good thermal stability, good electrochemical performance, and long lifespan	Nitta et al. (2015). [118]
	Use of LCO	Long discharge time, high specific energy, low power output	Shue et al. (2021). [119]
	NMC	High specific energy, high energy, relatively high capacity, high loading capacity, cost-saving, doesn't require built-in circuits.	Xiong S. (2019), Leal et al. (2023), Tallman and Takeuchi (2021). [128, 129, 130]
	NCA	High specific energy, decent specific power, large capacity, long lifecycle	Leal (2023), Tallman and Takeuchi, (2021), Yoshizawa and Ohzuku (2007). [129, 130, 140]
	LMO Or NMC with LTO anodes	Is extremely safe, has a long lifespan, and charges faster than other batteries.	
	NMO	Excellent rate capability, excellent cyclic stability, cost-effective, but limited cycle performance	Sun et al. (2018a), Sun et al. (2018b), and Fu et al. (2023) [135, 136, 137]
Separator	Use of polyolefin (PP, PE)-	Good mechanical strengthand chemical stability	(Zhang, Li, Yang, & Chen, 2021; Liu et al., 2020) [106, 110]
	Use of nanofiber (electrospun) separators with nanopore structures	Large specific surface area, small thickness, High porosity, good wettability with the electrolyte, high electrolytic absorption, high ionic conductivity, low mechanical strength	(Leng, Yang, Li, Arifeen, & Ko, 2022; Xing et al., 2022) [107, 111]
	Graphene oxides and their derivatives	Excellent mechanical, thermal, and electrical properties, and good electrolytic wettability	(Wang et al., 2019) [113]
	Graphene + Polyimide	Excellent thermal stability, good electrolyte absorption rates, Improved ionic conductivity, superior cycling efficiency and better C-rate discharge capacity	(Roh et al., 2022; Kang et al., 2022) [114, 115]
	Ceramic and polymer composites	Improved ion conductivity and thermal stability by suppressing thermal shrinkage, leading to safer battery.	
Electrolyte	LiBF_4_, LiPF_6_, LiAsF_6_ monohydrate, LiClO_4_, LiCF_3_SO_3_, LiN(CF_3_SO_2_)_2_ dissolved in carbonate solvents	dissociate and fully dissolve in non-aqueous media, allowing solvated ions to move in media at high mobility, highly resistant to oxidative decomposition at the cathode and inert.	Luo et al. (2021), Zhang & Ramadass (2012), [133, 189]

In spite of the improvements on the three key components—namely electrodes, separators, and electrolytes—other operational aspects affect the discharge and recharge time of an EV battery. These aspects include separator porosity, charge transfer, lithium deposition, electrode side reactions, and cycling frequency [190,191]. Sungvemmenla et al. (2022), Tomaszweska et al. (2019), Jiang et al. (2021), and Zhao and Li (2020) reported that separator porosity reduces over time in the battery lifetime. They suggest regenerating the separator when its porosity has reduced by 40 %. Furthermore, it has been reported elsewhere that the negative electrode affects recharging time more significantly than the positive electrode [145, 169, 170, 171].

## 9 Lithium plating

Metal deposition may result from an exceptionally high recharging and discharging rate. Li deposition is a common phenomenon due to lithium's high reactivity. During high-rate charging, the Li-ions may not be able to enter the anode quickly enough, causing them to plate out as metallic Li on the anode's surface [190]. This rapid deposition can significantly increase cell temperature, adversely affecting the battery's overall capacity, lifespan, and safety, and may also contribute to increased internal resistance. Such conditions can diminish the battery's efficiency and pose a safety hazard, as deposited metallic Li may form dendrites, potentially causing a short circuit, fire, or explosion [42,144]. When metallic Li is deposited onto the surface of the electrodes, it forms a layer that impacts on charging and discharging rates, resulting in reduced energy efficiency and heightened heat generation [13].

Considering the longevity and safe functioning of electric vehicle (EV) batteries involves carefully balancing the capacity between the cathode and anode, as this aspect is recognized as a crucial factor in cell design. A geometrically oversized area and a slight excess capacity of the anode relative to the cathode are desired for enhanced safety. This aims to reduce the occurrence of Li plating on the surface of the anode during the charging process, a critical event that results in accelerated ageing and a decline in safety [191].

Numerous efforts have been undertaken to enhance the speed of charging in EVs. These efforts involve techniques such as preheating or pre-warming up the cells to improve electrode kinetics and utilizing higher voltage systems to lower current levels. It is crucial to develop optimized charging algorithms that enable rapid charging without compromising the lifespan or safety of the battery, especially for future EVs [42]. In a study by Chen et al. (2019), silicon material/graphite was explored in a pouch cell, with the cathode being LiNi_0.8_Co_0.15_Al_0.05_O_2_ and the anode SiOx/Graphite. The study investigated varying N/P ratios between 0.85 and 1.8 to improve the energy density of Li-ion batteries. Electrochemical tests conducted in this study demonstrated that an N/P ratio closer to unity provides the optimal trade-off between energy density and cycle performance. The research emphasized the importance of studying the N/P ratio to understand the balance between initial coulombic efficiency and cycling stability. It was observed that substantial capacity loss during early cycles depletes active Li from the cathode, significantly impacting the long-term cycle stability across different N/P ratios. This effect could potentially lead to overcharging or over-discharging, hindering the practical use of silicon in commercial batteries [192].

It is important to note that the non-uniformity in the distribution of current is intensified and magnified when the current is increased. Under high charging rates, the anode's overpotential tends to decrease the potential levels to such an extent that Li plating can occur, effectively decreasing the N/P ratio of the battery below 1 [144]. Overpotential serves as a measure of the resistance Li-ions face when entering the anode. At high charge rates, this resistance can become significant, leading ions to plate out as metallic Li instead of entering the anode material. Consequently, this can reduce the N/P ratio below 1, indicating fewer lithium ions in the anode than in the cathode. Such a scenario can exacerbate problems with current distribution and result in more significant degradation and reduced battery performance over time [193]. The primary objective of maintaining an N/P ratio typically >1 is to minimize the potential for Li plating development. A low N/P ratio, corresponding to a high cathode-to-anode areal capacity ratio close to unity (1), is considered risky. This condition increases the likelihood of locally saturating the graphite anode with Li-ions, causing the local potential to drop below zero compared to Li^+^/Li. Such conditions can result in Li plating in batteries, posing potential risks [181,194].

**Fig. 5:**
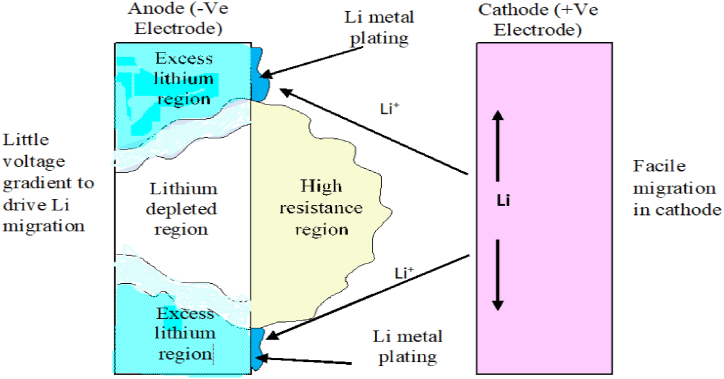
The illustration of the lithium plating formation process.

Consequently, the occurrence of Li plating, involving the reduction of Li-ions to metallic Li (Fig. 5), is particularly risky as it can lead to the formation of dendritic Li structures.

These Li dendrites, formed due to the uneven deposition of Li on the anode and cathode, pose risks of short circuits and capacity loss in batteries. Additionally, the presence of dendrites increases the likelihood of thermal runaway in the cell [195]. Moreover, the formation of metallic Li allows for the decomposition of the electrolyte. Since the solid SEI on metallic Li is unstable, the continuously generated Li, given its extremely reductive properties, gradually consumes the electrolytes and generates gaseous byproducts. The degradation of electrolytes compromises the cell's cycling stability, a concern that becomes apparent unless a short circuit occurs first in batteries [196].

The potential for Li plating during the charging process increases as the temperatures decrease, negatively affecting capacity retention. Charging batteries at low temperatures is associated with the occurrence of Li plating due to the slow Li-ion transit in the electrolyte, resulting in substantial overpotential at relatively low currents [43,197]. Cell characteristics, age, and C-rate are a few examples of variables that can affect the temperature threshold below which Li plating is likely to occur [197]. While some researchers have documented Li plating at temperatures lower than 25 °C [[197], [198], [199]], it is important to note that it can also occur at higher temperatures, especially with high-energy density cells, high C-rates, and an increase in Li-ion reduction kinetics [200,201]. Nevertheless, the correlation between Li plating and temperature holds true when the anode's potential is sufficiently low to permit Li plating thermodynamically. In other words, a higher operating temperature would likely accelerate the rate of dendrite formation in batteries if Li plating were to occur [202]. Minimizing Li plating is essential for the efficient operation of fast-charging equipment, which is often highly reliant on temperature, as stated above. Reported power conversion efficiencies of 50 kW chargers have reached up to 93 % at 25 °C but can drop to as low as 39 % when operating at −25 °C due to the derating of power levels by battery management systems (BMSs) at lower temperatures [203].

To minimize the risk of Li plating, BMSs in electric vehicles typically employ sophisticated algorithms to control charging and discharging rates while monitoring the battery's temperature and state of charge [204]. Additionally, battery manufacturers may use advanced electrode materials and electrolytes that are less prone to Li plating [204,205]. For instance, developing anode materials with higher lithiation potentials is crucial to mitigate the likelihood of Li deposition. One such material is spinel Li titanate (Li_4_Ti_5_O_12_), which exhibits a higher potential for Li insertion but offers a lower specific capacity (∼175 mA h g^−1^), reducing the energy density for practical applications of Li-ion batteries. Silicon and phosphorus have a very high specific capacity and a high lithiation potential, theoretically making them promising anode materials for future Li-ion battery systems. However, they face practical challenges due to significant lithiation volume expansion of up to 300 %. Nevertheless, progress has been made in implementing these materials in practical settings by utilizing nanometerization techniques [13].

Researchers should continue to explore and develop advanced anode materials, such as silicon and phosphorus, which offer higher lithiation potentials but face challenges due to volumetric expansion. Employing techniques like nanometerization may present viable solutions to these challenges. To further reduce the risk of Li plating, innovative designs in electrolytes and electrodes are needed to accommodate faster charging rates and higher energy densities while ensuring stability and safety. Since charging efficiency and safety are highly temperature-dependent, improving thermal management systems within the battery pack could prevent conditions that favour Li plating. Finally, the implementation of even more sophisticated algorithms that dynamically monitor and adjust charging and discharging rates based on real-time battery performance data will not only help prevent lithium plating but also extend the battery's operational lifespan.

## 10 Conclusions and future perspectives

### 10.1 Conclusions

In conclusion, optimizing the components of Li-ion batteries, including the anode electrode, cathode electrode, separator, and electrolyte, is crucial for enhancing overall performance. The diverse materials and composites discussed in this review paper present innovative approaches to improve specific parameters associated with each category. For anode electrode improvement, the use of carbon-based materials such as graphite, graphene, and graphene oxides, as well as various metal composites like TiO_2_, LTO, LMO, Co_3_O_4_, Fe_2_O_4_, NiO, and alloy composites like Si and Sn, have shown promise in maximizing cell energy density but are susceptible to Li plating. Additionally, novel structures like TiNb_2_O_7_ anodes and MoS_2_ with 2D and 3D structures demonstrate high theoretical capacity, quick Li^+^ intercalation/deintercalation, and excellent cycling stability. The cathode electrode benefits from a range of materials, each contributing unique advantages. LFP is notable for its good thermal stability, making it a valuable choice for cathode electrode development. LCO stands out with its longer discharge time and high specific energy. NMC brings the benefits of high specific energy, relatively high capacity, and the advantage of not requiring built-in circuits. On the other hand, NCA exhibits high specific energy, decent specific power, and a long lifecycle. Finally, LTO stands out as a safer option with a long lifespan, serving as a cathode material with the added benefit of faster charging compared to other batteries. The separator plays a crucial role in maintaining the mechanical strength and chemical stability of lithium-ion batteries. The use of polyolefin, electrospun nanofiber separators, graphene oxides, and graphene-polyimide composites addresses key factors such as mechanical strength, specific surface area, thickness, porosity, wettability, and ionic conductivity in Li-ion batteries. The use of ceramic and polymer composites as separators improves ion conductivity and thermal stability, thereby enhancing battery safety. In the electrolyte category, the selection of LiBF_4_, LiPF_6_, LiAsF_6_ monohydrate, LiClO_4_, LiCF_3_SO_3_, and LiN(CF_3_SO_2_)_2_ dissolved in carbonate solvents is pivotal. These electrolytes dissociate and fully dissolve in non-aqueous media, allowing solvated ions to move with high mobility. Moreover, they exhibit high resistance to oxidative decomposition at the cathode and remain inert, contributing to the overall efficiency and longevity of the battery. The formation of a stable and effective SEI is crucial for the performance and safety of EV batteries. A well-formed SEI layer can prevent the occurrence of Li plating, a major issue affecting the performance and lifespan of EV batteries. Various materials, such as metal composites, TiNb_2_O_7_ anodes, and polymer nanocomposites based on graphite and graphene, can be employed to improve the stability and efficiency of the SEI layer, thereby reducing the risk of Li plating and enhancing battery performance. Additionally, using metallic Li as an anode material can increase the risk of Li plating due to its high reactivity and low stability. However, using metallic Li with 3D structural matrices, specifically electrospun, can improve rate capability, facilitate faster Li-ion diffusion, ensure stable cycling, and minimize overpotential for Li plating or stripping. Overall, forming a stable SEI layer and preventing Li plating are important considerations in the design and development of EV batteries. The use of appropriate materials and technologies contributes to enhancing the performance (functional efficiency), safety, and lifespan of these batteries. In summary, the integration of these advanced materials and designs, as highlighted in this review paper, provides a comprehensive framework for improving Li-ion battery performance across multiple parameters. As battery technology continues to advance, the exploration of these diverse materials and their combinations holds great promise for developing high-performance, reliable, and safe Li-ion batteries.

### 10.2 Future perspective

Material selection and modification stand out as key areas for continued research, given that numerous materials and material designs have demonstrated favorable attributes, such as high energy density and capacity. These characteristics are exemplified in applications like fast-charging and prolonged discharge durations. Materials or structure strategies addressed in research have only had their performance evaluated on a laboratory scale, and they may not necessarily lead to enhancements when integrated into commercial battery designs and packs, warranting further research. Notably, the deficiency in ion conduction, particularly in polymer-based electrolytes, remains a gap that urgently needs addressing. The potential solution lies in adopting liquid electrolytes with diverse solvents and optimized dielectric and viscosity constants to enhance ion conduction rates. This necessitates a field-trial process, emphasizing the importance of consistent SEI formation, optimization studies, and the implementation of sophisticated operating techniques to foster an elevated rate of ion conduction. In the context of battery separators, advanced fiber materials and post-treatment processes are critical for ensuring commercial viability. Exploring controllable nanostructures in electrospun separators, such as core-shell or hollow structures, holds promise for innovative design approaches. Theoretical and experimental studies are equally vital for understanding and improving heat transfer properties within electrospun separators. Turning attention to cathodes, integrating machine learning algorithms in materials design and AI-assisted virtual experiments emerges as a future avenue to enhance electrochemical performance. While current nickel-rich and cobalt-free cathode materials face technical challenges, researchers are actively addressing these issues through material engineering, surface coating techniques, and leveraging computational models for prediction and optimization. This concerted effort aims to boost the overall performance and stability of cathode materials. Despite significant advancements in carbon-based anodes, particularly graphite materials, there remains an opportunity for extensive research to enhance magnification and current capacity. Future studies will prioritize understanding the dynamic behavior of carbon anode materials, considering economic and safety factors.

## Additional information

This review paper is adequately referenced; therefore, no additional information is required.

## CRediT authorship contribution statement

**Alex K. Koech:** Writing – review & editing, Writing – original draft. **Gershom Mwandila:** Writing – review & editing, Supervision. **Francis Mulolani:** Supervision.

## Declaration of competing interest

The authors declare that they have no known competing financial interests or personal relationships that could have influenced the work reported in this paper.

## References

[1] Abo-Khalil A.G., Abdelkareem M.A., Sayed E.T., Maghrabie H.M., Radwan A., Rezk H., Olabi A. (2022). Electric vehicle impact on energy industry, policy. technical barriers, and power systems..

[2] Tomaszewska A., Chu Z., Feng X., Kane S.O., Liu X., Chen J., Marinescu M. (2019). Lithium-ion battery fast charging: a review. eTransportation.

[3] Mohammad I., Blondeau L., Leroy J., Khodja H., Gauthier M. (2021).

[4] Zhao G., Wang X., Negnevitsky M. (2022). Connecting battery technologies for electric vehicles from battery materials to management. Review.

[5] Rajper S.Z., Albrecht J. (2020). Prospects of electric vehicles in the developing countries. Lit. Rev..

[6] Chen L., Msigwa G., Yang M. (2022). “Strategies to achieve a carbon neutral society: a review. Environ. Chem. Lett..

[7] Koech K.A., Kumar A., Siagi Z.O. (2020). In situ transesterification of spirulina microalgae to produce biodiesel using microwave irradiation. J. Energy.

[8] Yang Z., Huang H., Lin F. (2022). Sustainable electric vehicle batteries for a sustainable world: perspectives on battery cathodes, environment, supply chain, manufacturing, life cycle, and policy. Adv. Energy Mater..

[9] Alanazi F. (2023). Electric vehicles: benefits, challenges, and potential solutions for widespread adaptation.

[10] Shah A., Shah K., Shah C., Shah M. (2022). State of charge, remaining useful life and knee point estimation based on artificial intelligence and Machine learning in lithium-ion EV batteries. A comprehensive review.

[11] Ali Z.M., Calasan M., Gandoman F.H., Jurado F., Aleem S.H. (2023). Review of batteries reliability in electric vehicle and E-mobility applications.

[12] Cheng Y., Kang Y., Zhao Y., Wang L., liu J., li Y., li b. (2021). A review of lithium-ion battery safety concerns. The issues, strategies, and testing standards.

[13] Lipu M.S., Miah M.S., Hasan K., Meraj S.T., al e. (2022). Battery management, key technologies methods, issues, and future trends of electric vehicles; A pathway toward achieving sustainable. Development Goals.

[14] How D., Hannan M., Hossain L.M., Ker P. (2019). State of charge estimation for lithium-ion batteries using model-based and data-driven methods. Review.

[15] Hannan M., Hoque M., Hussain A., Yusof Y., Ker P. (2018). State-of-the-Art and energy management system of lithium-ion batteries in electric vehicle applications: issues and recommendations. IEEE Acess.

[16] Zubi G., Dufo-López R., Carvalho M., Pasaoglu G. (2018). The lithium-ion battery: state of the art and future perspectives. Renew. Sustain. Renew. Sustain. Energy Rev..

[17] Zhang R., Xia B., Li B., Cao L., Lai Y., Zheng W., al e. (2018). State of the art of lithium-ion battery SOC estimation for electrical vehicles. Energies.

[18] Liu W., placke T., Chau K. (2022). Overview of batteries and battery management for electric vehicles- A. Review.

[19] Sriram V.K., Michael L., Hungund S., Fernades M. (2022). Factors influencing adoption of electric vehicles – a case in India.

[20] Altenburg Tilman (2014).

[21] Hossain M.S., Kumar L., Assad M.E., Alayi R. (2022). Advancements and future prospects of electric vehicle technologies: a comprehensive review.

[22] Deng J., Bae C., Denlinger A., Miller T. (2020). Electric vehicles batteries. Requirements and Challenges-A Commentary.

[23] Mastoi M.S., Zhuang S., Munir H.M., Haris M., Hassan M., Usman M., Ro J.-S. (2022). An in-depth analysis of electric vehicle charging station infrastructure. policy implications, and future trends - Review article.

[24] Sanguesa J., Torres-Sanz V., Garrido P., Martinez F., Maquerz-Barja J. (2021).

[25] Pipitone E., Caltabellotta S., Occhipinti L. (2021). A life cycle environmental impact comparison between traditional. Hybrid, and Electric Vehicles in the European Context.

[26] Jannesar Niri Anahita, Poelzer Gregory A., Zhang Steven E., Rosenkranz Jan, Pettersson Maria, Ghorbani Yousef (2024). “Sustainability challenges throughout the electric vehicle battery value chain,”. Renew. Sustain. Energy Rev..

[27] Irle Roland (2023). “Global EV sales for 2022. https://www.ev-volumes.com/country/total-world-plug-in-vehicle-volumes/.

[28] Marcus Lu (2024).

[29] IEA (2023). https://www.iea.org/reports/global-ev-outlook-2023.

[30] Neil King (2023).

[31] Sachs Goldman (2023).

[32] Lindwall Courtney (2022).

[33] Wang W., Wei X., Choi D., Lu X., Yang G., Sun C. (2015).

[34] Czerwinski F. (2021).

[35] Grant P.S., Greenwood D., Pardikar K., Smith R., Entwistle T., Middlemiss L.A., Capener M.J. (2022). Roadmap on Li-ion battery manufacturing research. Journal of Physics Energy.

[36] Dufo-López R., Cortés-Arcos T., Artal-Sevil J.S., Bernal-Agustín J.L. (2021). Comparison ofLead-acid and Li-ion BatteriesLifetime prediction models in stand-alone photovoltaic systems. Appl. Sci..

[37] Jeffrey Wishart (2014).

[38] Un-Noor Fuad, Sanjeevikumar P., Mihet-Popa Lucian, Molan Mohammad, Hossain Eklas (2017). “A comprehensive study of key electric vehicle (EV) components, technologies, challenges, impacts, and future direction of development,”. Energies.

[39] Morris Brenna, Foiadelli Federica, Leone Carola, Longo Michela (2020). Electric vehicles charging technology review and optimal size estimation. Journal of Electrical Engineering & Technology.

[40] Chakraborty P., Parker R., Hoque T., Cruz J., Du L., Wang S., al e. (2022).

[41] Ren D., Lu L., Shen P., Feng X., Han X., Ouyang M. (2019). Battery remaining discharge energy estimation based on prediction of future operating conditions.

[42] Lebrouhi B.E., Khattari Y., Lamrani B., Maaroufi M., Zeraouli Y., Kousksou T. (2021). Key challenges for a large-scale development of battery electric vehicles: a comprehensive review. J. Energy Storage.

[43] Ma S., Jiang M., Tao P., Song C., Wu J., Wang J., Shang W. (2018). Temperature effect and thermal impact in lithium-ion batteries: a review. Prog. Nat. Sci.: Mater. Int..

[44] Trahey Lynn, Brushett Fikile R., Balsara Nitash P., Ceder Gerbrand, Cheng Lei, Chiang Yet-Ming, Hahn Nathan T., Ingram Brian J., Minteer Shelley D., Moore Jeffrey S., Mueller Karl T., Nazar Linda F., Persson Kristin A., Siegel Donald J., Xu Kang, Zavadil Kevin R., Srinivasan Venkat, Crabtree George W. (2020). ”Energy storage emerging: a perspective from the JointCenter for energy storage research,”.

[45] Tarascon J.M. (2016). The Li-ion battery: 25 Years of exciting and enriching experiences. Interface magazine.

[46] Sun Jiaxun, Ye Lingqian, Zhao Xinran, Zhang Peipei, Yang Jun (2023). “Electronic modulation and structural engineering of carbon-based anodes for low-temperature lithium-ion batteries. Review.

[47] Zhang Jing, Qiao Jinshuo, Sun Kening, Wang Zhenhua (2022). Balancing particle properties for practical lithium-ion batteries. Particuology.

[48] Alipour Mohammad, Ziebert Carlos, Conte Fiorentino Valerio, Kizilel Riza (2020). “A review on temperature-dependent electrochemical properties. Aging, and Performance of Lithium-Ion Cells,”.

[49] Weiss Manuel, Ruess Raffael, Kasnatscheew Johannes, Levartovsky Yehonatan, Levy Natasha Ronith, Minnmann Philip, Stolz Lukas, Waldmann Thomas, Aurbach Doron, Winter Martin, Ein-Eli Yair, Janek Jürgen (2021). “Fast charging of lithium‐ion batteries:. A Review of Materials Aspects,”.

[50] Chabot Victor, Farhad Siamak, Chen Zhongwei, Fung Alan S., Yu Aiping, Hamdullahpur Feridun (2013). Effect of electrode physical and chemical properties on lithium-ion battery performance.

[51] Sharova Varvara (2018). “Enhancing the performance of lithium batteries through the development of improved electrolyte formulation, formation protocol and graphite surface modification,”. (Doctoral dissertation, Dissertation, Karlsruhe, Karlsruher Institut für Technologie (KIT), 2017).

[52] Zhao Rui, Zhang Sijie, Gu Junjie, Liu Jie (2016). Modeling the electrochemical behaviors of charging Li-ion batteries with different initial electrolyte salt concentrations. nt. J. Energy Res..

[53] Sarkar Susmita, Hoffmann John Alexander, Park Jonghyun (2021). Micro-macroscopic modeling of a lithium-ion battery by considering grain boundaries of active materials. Electrochim. Acta.

[54] Ye Zhixin, Zou Zhimin, Jiang Chunhai (2023). Enhancing the rate and cycle performance of graphite anode for Li-ion batteries by constructing a multidimensional conducting network. Diam. Relat. Mater..

[55] Kang Jianqiang, Gu Li, Wang Jing V., Wu Zhixuan, Zhu Guorong, Li Zhe (2022). Blending fiber-shaped long-range conductive additives for better battery performance: Mechanism study based on heterogeneous electrode model. J. Power Sources.

[56] Jiao Xingxing, Kirianova Alina V., Xu Xieyu, Kapitanova Olesya O., Krivchenko Victor A., Napolskiy Filipp S., Volkov Valentyn S., Gallyamov Marat O., Liu Yangyang (2023). Conductive additives for improving the rate capability of cathode materials in secondary lithium batteries. ACS Appl. Energy Mater..

[57] Mizuno Fuminori, Hayashi Akitoshi, Tadanaga Kiyoharu, Tatsumisago Masahiro (2005). Effects of conductive additives in composite positive electrodes on charge-discharge behaviors of all-solid-state lithium secondary batteries. J. Electrochem. Soc..

[58] Rehnlund David, Wang Zhaohui, Nyholm Leif (2022). “Lithium-Diffusion induced capacity losses in lithium-based batteries. Review.

[59] Zhu Yaqi, Li Jie, Sadeq Saleh M., Pham Hiep, Patwary Plateau Tazdik, Panat Rahul, Park Jonghyun (2020). “Towards high-performance Li-ion batteries via optimized three-dimensional micro-lattice electrode architectures. J. Power Sources.

[60] Gao Xinran, Xing Zheng, Wang Mingyue, Nie Chuanhao, Shang Zhichao, Bai Zhongchao, Dou Shi Xue, Wang Nana (2023). “Comprehensive insights into solid-state electrolytes and electrode-electrolyte interfaces in all-solid-state sodium-ion batteries. Energy Storage Mater..

[61] Li Q., Chen J., Fan L., Kong X., Lu Y. (2016). Progress in electrolytes for rechargeable Li-based batteries and beyond. Review.

[62] Kalpana R.N., Dhoble S.J. (2021).

[63] Schaefer J., Lu Y., Moganty S., Agarwal P., Jayaprakash N., Archer L. (2012). Electrolytes for high-energy lithium batteries - a Review. Appl. Nanosci..

[64] Luo D., Li M., Zheng Y., Ma Q., Gao R., Zhang Z., Dou H., Wen G., Shui L., Yu A., Wang X., Chen Z. (2021). Electrolyte design for lithium metal anode-based batteries toward extreme temperature application.

[65] Nagde K.R., Dhoble S.J. (2021). Li-S ion batteries: a substitute for Li-ion storage batteries. Energy Materials.

[66] Nizam Uddin Khan F.M., Rasul Mohammad G., Sayem A.S.M., Mandal Nirmal (2023). Maximizing energy density of lithium-ion batteries for electric vehicles: a critical review. Energy Rep..

[67] Yang Li, Ravdel Boris, Lucht Brett L. (2010). Electrolyte reactions with the surface of high voltage LiNi 0.5Mn1.5O 4 cathodes for lithium-ion batteries. Electrochem. Solid State Lett..

[68] Qi Li, Chen Juner, Fan Lei, Kong Xueqian, Lu Yingying (2016). Progress in electrolytes for rechargeable Li-based batteries and beyond. Green Energy Environ..

[69] Zhang Zhengcheng, Hu Libo, Wu Huiming, Weng Wei, Koh Meiten, Redfern Paul C., Curtiss Larry A., Amine Khalil (2013). “Fluorinated electrolytes for 5 V lithium-ion battery chemistry,”.

[70] Lavi Ortal, Luski Shalom, Shpigel Netanel, Menachem Chen, Pomerantz Zvika, Elias Yuval, Aurbach Doron (2020). Electrolyte solutions for rechargeable Li-ion batteries based on fluorinated solvents. ACS Appl. Energy Mater..

[71] Fan Xiulin, Ji Xiao, Chen Long, Chen Ji, Deng Tao, Han Fudong, Yue Jie, Piao Nan, Wang Ruixing, Zhou Xiuquan, Xiao Xuezhang, Chen Lixin, Wang Chunsheng (2019). All-temperature batteries enabled by fluorinated electrolytes with non-polar solvents. Nat. Energy.

[72] Kerner Manfred, Lim Du-Hyun, Jeschke Steffen, Rydholm Tomas, Ahn Jou-Hyeon, Scheers Johan (2016). Towards more thermally stable Li-ion battery electrolytes with salts and solvents sharing nitrile functionality. J. Power Sources.

[73] Lin Ziyang, Wang Zhuofan (2023). Application of solid polymer electrolytes for solid-state sodium batteries. MATEC Web of Conferences.

[74] Cheng Zhiwei, Tong Liu, Zhao Bin, Shen Fei, Jin Haiyun, Han Xiaogang (2021). Recent advances in organic-inorganic composite solid electrolytes for all-solid-state lithium batteries. Energy Storage Mater..

[75] Yang Hui, Wu Nianqiang (2022). Ionic conductivity and ion transport mechanisms of solid-state lithium-ion battery electrolytes: a review.

[76] Kautz David J., Cao Xia, Gao Peiyuan, Matthews Bethany E., Xu Yaobin, Han Kee Sung, Omenya Fredrick, Engelhard Mark H., Jia Hao, Wang Chongmin, Zhang Ji-Guang, Xu nd Wu (2023). Designing electrolytes with controlled solvation structure for fast-charging lithium-ion batteries. Adv. Energy Mater..

[77] Logan Eric, Dahn J.R. (2020). Electrolyte design for fast-charging Li-ion batteries. Trends in Chemistry.

[78] Zhao Wengao, Si Mayan, Wang Kuan, Brack Enzo, Zhang Ziyan, Fan Xinming, Battaglia Corsin (2023). Electrolyte optimization to improve the high-voltage operation of single-crystal LiNi_0.83_Co_0.11_Mn_0.06_O_2_ in lithium-ion batteries.

[79] Zhou Li, Zhang Miao, Huo Yani, Bai Liping, He Suhang, Wang Jinying, Jia Chuancheng, Guo Xuefeng (2024). Application of ionic liquids in single-molecule junctions: recent advances and prospects. Green Energy Environ..

[80] Wang A., Kadam S., Li H., shi S., Oi Y. (2018). Review on modeling of the anode solid electrolyte interphase (SEI) for lithium-ion batteries.

[81] Xu L., Tang S., Cheng Y., wang K., Liang J., Liu C., Mai L. (2018). Interfaces in solid-state lithium batteries. Review.

[82] Rao R., Hu J., Lee P.-H. (2022).

[83] Lundström Robin, Gogoi Neeha, Melin Tim, Berg Erik J. (2024). Unveiling reaction pathways of ethylene carbonate and vinylene carbonate in Li-ion batteries. J. Phys. Chem..

[84] Heiskanen S.K., Kim J., Lucht B.L. (2019). Generation and evolution of the olid electrolyte interphase of lithium-ion batteries.

[85] An S.J., Li J., Daniel C., Mohanty d., Nagpure S., III D.l. (2016). The state of understanding of the lithium-ion-battery graphite solid electrolyte interphase (SEI) and its relationship to formation cycling-A review.

[86] Adenusi H., Chass G.A., Passerini S., Tian K.V., Chen G. (2023). Lithium batteries and the solid electrolyte interphase (SEI)—progress and outlook - a review.

[87] Beheshti S.H., Javanbakht M., Omidvar H., Hosen M.S., Hubin A., Mierlo J.V., Berecibar M. (2022). Development, retainment, and assessment of the graphite-electrolyte interphase in Li-ion batteries regarding the functionality of SEI-forming additives-. Review.

[88] Lin D., Liu Y., Cui Y. (2020). 1. "Solid electrolyte interphase in Li-ion batteries: an overview of its composition, formation, causes, and mitigation strategies". Nano Energy.

[89] Manthiram A., X Y. (2019). Solid electrolyte interphase (SEI) formation and mitigation on high energy lithium-ion batteries. Adv. Energy Mater..

[90] Nie Z., Zhou J., Chen J. (2018). The role of solid electrolyte interphase in electrochemical performance and degradation of high-energy-density lithium-ion batteries. Angew. Chem. Int. Ed..

[91] Gordin M.L., Song D., Wang C. (2015). Stability of the solid electrolyte interphase on lithium-ion batteries: a review. ECS Journal of Solid State Science and Technology.

[92] Park M., Yu S., Jin Y. (2018). Solid electrolyte interphase (SEI) formation and its role in high-performance lithium-ion batteries. Journal of Electrochemical Energy Conversion and Storage.

[93] Kolzenberg L.v., Latz A., Horstmann B. (2020). Solid–electrolyte interphase during battery cycling: theory of growth regimes. National Center for Biotechnology.

[94] Li M., Feng M., Luo D., Chen Z. (2020). Fast charging Li-ion batteries for a new era of electric vehicles. Cell Report Physical Science.

[95] Mo R., Li F., Tan X., Xu P., Tao R., Shen G. (2019).

[96] Ma S., Jiang M., Tao P., Song C., Wu J., Wang J., Shang W. (2018). Temperature effect and thermal impact in lithium-ion batteries: a review. Prog. Nat. Sci.: Mater. Int..

[97] Xia S., Wu X., Zhang Z., Cui Y., Liu W. (2019). Practical challenges and future perspectives of all-solid-state lithium-metal batteries. Review.

[98] Peled E., Menkin S. (2017). Review—SEI: past. Present and Future.

[99] McBrayer J., Apblett C., Harrison K., Fenton K., Minter S. (2021).

[100] Li L., Dai H., Wang C. (2021). Electrolyte additives: adding the stability of lithium metal anodes - a Review. Wiley.

[101] Meng J., Jia G., Yang H., Wang M. (2022). Recent advances for SEI of hard carbon anode in sodium-ion batteries: a mini review. Front. Chem..

[102] Zhang X., Meng J., Wang X., Xiao Z., Wu P., Mai L. (2021).

[103] Li H., Han Y., Liu S. (2020). 1. "Solid electrolyte interphase (SEI) engineering in high-performance lithium-ion batteries". Adv. Sci..

[104] Aurbach D., Lu Z., Schechter A. (2016). Recent advances in the understanding of solid electrolyte interphase (SEI) formation and its role in Li-ion batteries. Electrochim. Acta.

[105] Cheng L., Zhang J. (2014). Solid electrolyte interphase in lithium-ion batteries: mechanisms and properties. J. Power Sources.

[106] Zhang L., Li X., Yang M., Chen W. (2021).

[107] Leng X., Yang M., Li C., Arifeen W.U., Ko T.J. (2022). High-performance separator for lithium-ion battery based on dual-hybridizing of materials and processes.

[108] Lin W., Wang F., Wang H., Li H., Fan Y., Chan D., Zhang Y. (2022). Thermal-stable separators: design principles and strategies towards safe lithium-ion battery operations.

[109] Zhou Y.-T., Yang J., Liang H.-Q., Pi J.-K., Zhang C., Xu Z.-K. (2018).

[110] Liu Z., Jiang Y., Hu Q., Guo S., Yu L., Li Q., Huo X. (2020). Safer lithium-ion batteries from the separator aspect: development and future perspectives - a review. Energy & Environmmental Material.

[111] Xing J., Li J., Fan W., Zhao T., Chen X., Li H., Zhao Y. (2022). A review on nanofibrous separators towards enhanced mechanical properties for lithium-ion batteries. Compos. B Eng..

[112] Nauman S., Lubineau G., Aharbi H.L. (2021). Post processing strategies for the enhancement of mechanical properties of ENMs (electrospun nanofibrous membranes). Review.

[113] Wang L., Liu F., Shao W., Cui S., Zhao Y., Zhou Y., He J. (2019).

[114] Roh Y., Jin D., Kim E., Byun S., Lee Y.-S., Ryou M.-H., Lee Y.M. (2022). Highly improved thermal stability of the ceramic coating layer on the polyethylene separator via chemical crosslinking between ceramic particles and polymeric binders. Chem. Eng. J..

[115] Kang J., Yan Z., Gao L., Zhang Y., Liu W., Yang Q., Kang W. (2022). Improved ionic conductivity and enhancedinterfacial stability of solid polymer electrolytes with porous ferroelectric ceramic nanofibers. Energy Storage Mater..

[116] Ahmed F.E., Lalia B.S., Hashaikeh R. (2015). A review on electrospinning for membrane fabrication: challenges and applications.

[117] Ma X., Praveen K., Yang R., Wang Z. (2017). Electrospun polyacrylonitrile nanofibrous membranes with varied fiber diameters and different membrane porosities as lithium-ion battery separators.

[118] Nitta N., Wu F., Lee J.T., Yushin G. (2015). Li-ion battery materials: present and future. Mater. Today.

[119] Shu X., Guo Y., Yang W., Wei K., Wui G. (2021). Life-cycle assessment of the environmental impact of the batteries used in pure electric passenger cars. Energy Rep..

[120] Mahmud S., Rahman M., Kamruzzaman M., Ali M.O., Emon M.s., Khatun H., Ali M.R. (2022). Recent advances in lithium-ion battery materials for improved electrochemical performance: a review. Results in Engineering.

[121] Liu S., Wang B., Zhang X., Zhao S., Zhang Z., Yu H. (2021). Reviving the lithium-manganese-based layered oxide cathodes for lithium-ion batteries - review. Matter.

[122] Sun W., Liu H., Liu Y., Bai Q., Liu W., Guo S., Zhao X.-Z. (2015). A general strategy to construct uniform carbon-coated spinel LiMn2O4 nanowires for ultrafast rechargeable lithium-ion batteries with a long cycle life.

[123] Zheng X., Cai Z., Sun J., He J., Rao W., Wang J., Zhou C. (2023). Nickel-rich layered oxide cathodes for lithium-ion batteries: failure mechanisms and modification strategies.

[124] Zeng X., Zhan C., Lu J., Amine K. (2018). Stabilization of a high-capacity and high-power nickel-based cathode for Li-ion. Batteries- A review.

[125] Wei H.-x., Huang Y.-d., Tang L.-b., Yan C., He Z.-j., Mao J., Zheng J.-c. (2021). Lithium-rich manganese-based cathode materials with highly stable lattice and surface enabled by perovskite-type phase-compatible layer.

[126] Hlongwa N.W., Raleie N. (2022). Lithiated manganese-based materials for lithium-ion capacitor. Review.

[127] Chu B., Guo Y.-J., Shi J.-L., Yin Y.-X., Huang T., Su H., Li Y. (2022). Cobalt in high-energy-density layered cathode materials for lithium ion batteries. Review.

[128] Xiong Shihui (2019). “A study of the factors that affect lithium ion degradation,”. Doctoral dissertation, University of Missouri-Columbia).

[129] Leal V.M., Ribeiro J.S., Coelho E.L.D., Freitas M.B.J.G. (2023). Recycling of spent lithium-ion batteries as a sustainable solution to obtain raw materials for different applications. J. Energy Chem..

[130] Tallman K.R., Takeuchi E.S. (2021). Nickel-rich nickel manganese cobalt (NMC622) cathode lithiation mechanism and extended cycling effects using operando X-ray absorption spectroscopy. J. Phys. Chem. C.

[131] Yang H., Li L., Liu C., Chen J., Xia L., Liu Z., Duan J. (2020). Simultaneous synthesis and synergetic stabilization of Zr-doped and Li6Zr2O7-coated Ni-rich layered cathode for advanced lithium ion batteries.

[132] Chen S., Zhang X., Xia M., Wei K., Zhang L., Zhang X., Shu J. (2021).

[133] Luo Y.-h., Wei H.-x., Tang L.-b., Huang Y.-d., Wang Z.-y., He Z.-j., Zheng J.-c. (2022).

[134] Song L., Du J., Xiao Z., Jiang P., Cao Z., Zhu H. (2020). Research progress on the surface of high-nickel nickel–cobalt–manganese ternary cathode materials. Min. Rev..

[135] Sun W., Li Y., Liu Y., Guo Q., Luo S., Yang J., Xie K. (2018). Hierarchical waxberry-like LiNi0.5Mn1.5O4 as an advanced cathode material for lithium-ion batteries with a superior rate capability and long-term cyclability.

[136] Sun W., Li Y., Xie K., Luo S., Bai G., Tan X., Zheng C. (2018). Constructing hierarchical urchin-like LiNi0.5Mn1. 5O4 hollow spheres with exposed {111} facets as advanced cathode material for lithium-ion batteries.

[137] Fu T., Lu D., Yao Z., Li Y., Luo C., Yang T., Sun W. (2023). Advances in modification methods and the future prospects of high-voltage spinel LiNi0. 5Mn1.5O4 — a review.

[138] Noerochim L., Suwarno S., Idris N.H., Dipojono H.K. (2021). Recent development of nickel-rich and cobalt-free cathode materials for lithium-ion batteries.

[139] Kireeva N., Pervov V.S., Tsivadze A.Y. (2024). Machine learning-based evaluation of functional characteristics of Li-rich layered oxide cathode materials using the data of XPS and XRD spectra.

[140] Yoshizawa H., Ohzuku T. (2007). An application of lithium cobalt nickel manganese oxide to high-power and high-energy density lithium-ion batteries.

[141] Ali Z.M., Calasan M., Gandoman F.H., Jurado F., Aleem S.H. (2023). Review of batteries reliability in electric vehicle and E-mobility applications.

[142] Kouihen F.E., Kharbouch Z., Faik A. (2023). Review—advancements in synthesis methods for nickel-rich NCA cathode materials: optimizing synthesis conditions and their impact on electrochemical performances for next-generation lithium. Batteries.

[143] Fan Tianju, Wang Yujie, Krishna Harika Villa, Nimkar Amey, Wang Kai, Liu Xiaolang, Wang Meng, Xu Leimin, Elias Yuval, Sclar Hadar, Chae Munseok S., Min Yonggang, Lu Yuhao, Shpigel Netanel, Aurbach Doron (2022). Highly stable 4.6 V LiCoO2 cathodes for rechargeable Li batteries by rubidium‐based surface modifications. PMCID.

[144] Irfal Saaid Farish, Kasim Muhd Firdaus, Winie Tan, Elong Kelimah Anak, Azahidi Azira, Basri Nurul Dhabitah, Yaakob Muhamad Kamil, Mastuli Mohd Sufri, Shaffee Siti Nur Amira, Zolkiffly Mohd Zaid, Mahmood Mohamad Rusop (2024). Ni-rich lithium nickel manganese cobalt oxide cathode materials: a review on the synthesis methods and their electrochemical performances. Heliyon.

[145] Zhang Ruihan, Meng Zifei, Ma Xiaotu, Chen Mengyuan, Chen Bin, Zheng Yadong, Yao Zeyi, Vanaphuti Panawan, Bong Sungyool, Yang Zhenzhen, Wang Yan (2020). Understanding fundamental effects of Cu impurity in different forms for recovered LiNi0.6Co0.2Mn0.2O2 cathode materials. Nano Energy.

[146] Wu Tong, Wang Guange, Liu Borui, Huang Qing, Su Yuefeng, Wu Feng, Kelly Ryan (2021). The role of Cu impurity on the structure and electrochemical performance of Ni-rich cathode material for lithium-ion batteries. J. Power Sources.

[147] Wu Borong, Ren Yonghuan, Ning Li (2011).

[148] Pigłowska M., Kurc B., Galinski M., Fuc P., Kaminska M., Szymlet N., Daszkiewicz P. (2021). Challenges for safe electrolytes applied in lithium-ion cells. Review.

[149] Nzereogu P.U., Omah A.D., Ezema F.I., Iwuoha E.I., Nwanya A.C. (2022). Anode materials for lithium-ion batteries: a review. Applied Surface Science Advances.

[150] Deng R., He T. (2023). Flexible solid-state lithium-ion batteries. Mater. Struct..

[151] Chen J. (2013). Recent progress in advanced materials for lithium ion batteries. Materials.

[152] Kim D.S., Kim Y.E., Kim H. (2019).

[153] Dong C., Dong W., Lin X., Zhao Y., Li R., Huang F. (2020). Recent progress and perspectives of defective oxide anode materials for advanced lithium ion battery.

[154] Wu W., Wei Y., Chen H., Wei K., Li Z., He J., Yang H. (2021). In-situ encapsulation of α-Fe2O3 nanoparticles into ZnFe2O4 micro-sized capsules as high-performance lithium-ion battery anodes.

[155] Mohan V.B., Lau K.-t., Hui D., Bhattacharyya D. (2018). Graphene-based materials and their composites: a review on production, applications and product limitations. Compisites Part B: Engineering.

[156] Khan I., Saeed K., Khan I. (2019). Nanoparticles: properties, applications and toxicities. Arab. J. Chem..

[157] Wu Z.-S., Ren W., Xu L., Li F., Cheng a.H.-M. (2011). Doped graphene sheets as anode materials with superhigh rate and large capacity for lithium ion batteries.

[158] Yang Y., Li J., Chen D., Fu T. (2016). Binder-free carbon-coated Si/rGO nanocomposite electrode prepared by electrophoretic deposition as a high performance anode for lithium ion battery.

[159] Yu S., Guo B., Zeng T., Yang J., Bai J. (2022). Graphene-based lithium-ion battery anode materials manufactured by mechanochemical ball milling process. A review and perspective..

[160] Anasori B., Gogotsi Y. (2019). Introduction to 2D transition metal carbides and nitrides (MXenes).

[161] Li S., Cao X., Schmidt C.N., Xu Q., Uchaker E., Pei Y. (2016). TiNb2 O7/graphene composites as high-rate anode. J. Mater. Chem..

[162] Yun Q., Ge Y., Chen B., Li L., Wa Q., Long H., Zhang H. (2022).

[163] Ding J., Zhou Y., Li Y., Gao S. (2016). MoS2 nanosheet assembling superstructure with three-dimensional ion accessible site: a new class of bifunctional material for battery and electrocatalysis. Chem. Mater..

[164] Perera A., Madhushani K., Punchihewa B.T., Kumar A. (2023). MXene-based nanomaterials for multifunctional applications - a review. R.K. MXene-Based Nanomaterials for.

[165] Boaretto N., Garbayo I., Valiyaveettil-SobhanRaj S., Quintela A., Li C., Casas-Cabanas M., Aguesse F. (2021). Lithium solid-state batteries: state-of-the-art and challenges for materials, interfaces and processing. J. Power Sources.

[166] Li S., Wang K., Zhang G., Li S., Xu Y., Zhang X., Ma Y. (2022). Fast charging anode materials for lithium-ion batteries: current status and perspectives - a review.

[167] Guo R., Han W. (2022).

[168] Liu S., Wang X., Xie D., Xia X., Gu C., Wu J., Tu J. (2018).

[169] Qian J., Henderson W.A., Xu W., Bhattacharya P., Engelhard M., Borodin O., Zhang J.-G. (2015).

[170] Griffith K., Wiaderek K., Cibin G., Marbella L., Grey C. (2018). Niobium tungsten oxides for high-rate lithium-ion energy storage.

[171] Zhang L., Zeng M., Whu D., Yang X. (2019). Magnetic field regulating the graphite electrode for excellent lithium-ion batteries performance. ACS Sustain. Chem. Eng..

[172] Li L., Erb R.M., Wang J., Wang J., Chiang Y.-M. (2019).

[173] Fang M.-D., Ho T.-H., Yen J.-P., Lin Y.-R., Hong J.-L., Wu S.-H., Jow J.-J. (2015). Preparation of advanced carbon anode materials from mesocarbon microbeads for use in high C-rate lithium ion batteries.

[174] Sungvemmenla K.V.S., Soni C.B., Kumar V., Seh Z.W. (2022). Understanding the cathode–electrolyte interphase in lithium-ion batteries.

[175] Jiang F.-N., Yang S.-J., Liu H., Cheng X.-B., Liu L., Xiang R., Huang J.-Q. (2021). Mechanism understanding for stripping electrochemistry of Li metal anode - a review.

[176] Zhao D., Li S. (2020).

[177] Cao W., Li Q., Yu X., Li H. (2022). Controlling Li deposition below the interface. eScience.

[178] Tian Honghong, Graczyk-Zajac Magdalena, Kessler Alois, Weidenkaff Anke, Riedel Ralf (2023). “Recycling and reusing of graphite from retired lithium-ion batteries. Review.

[179] Hewathilake Sasanka, Karunarathne Niroshan, Wijayasinghe Athula, Balasooriya N.W.B., Arof A.K. (2017). Performance of developed natural vein graphite as the anodematerial of rechargeable lithium ion batteries. Ionics, Springer Nature.

[180] Lin Sun, Liu Yanxiu, Shao Rong, Wu Jun, Jiang Ruiyu, Zhong Jin (2022). Recent progress and future perspective on practical silicon anode-based lithium ion batteries. Energy Storage Mater..

[181] Li M., Wang C., Chen Z., Xu K., Lu J. (2020).

[182] Xing X., Li Y., Wang X., Petrova V., Liu H., Liu P. (2019).

[183] Yang Z., Charalambous H., Trask S.E., Montoya A., Jansen A., Wiaderek K.M., Bloom I. (2020).

[184] Colclasure A.M., Tanim T., Jansen A.N., Trask S.E. (2020).

[185] Kang, S. P.-Y.-H T. (2020). Thermal analysis of a parallel-configured battery pack (1S18P. Using 21700 Cells for a Battery-Powered Train.

[186] Zhang H., Liu X., Li H., Hasa I., Passerini S. (2020). Challenges and strategies for high-energy aqueous electrolyte rechargeable batteries.

[187] Richey F.W., Mccloskey B., Luntz A.C. (2016). Mg anode corrosion in aqueous electrolytes and implications for Mg-air batteries. J. Electrochem. Soc..

[188] Li J., Li H., Ma X., Stone W., Glazier S., Logan E., Dahn J.R. (2018). Methyl acetate as a Co-solvent in nmc532/graphite cells. J. Electrochem. Soc..

[189] Zhang Z., Ramadass P., Meyers R.A. (2012). Encyclopedia of Sustainability Science and Technology.

[190] Janakiraman U., Garrick T.R., Fortier M.E. (2020). Review—lithium plating detection methods in Li-ion batteries. J. Electrochem. Soc..

[191] Wu X., Song K., Zhang X., Hu N., Li L., Li W., Zhang H. (2019).

[192] Chen Z., Zhang I., Wu X., Song K., Ren B., Li T., Zhang S. (2019). Effect of N/P ratios on the performance of LiNi0.8Co0.15Al0.05O2||SiOx/Graphite lithium-ion batteries. J. Power Sources.

[193] Carnovale A., Li X. (2020). A modeling and experimental study of capacity fade for lithium-ion batteries. Elselvier.

[194] Kim C.S., Jeong K.M., Kim K., Yi C.W. (2015). Effects of capacity ratios between anode and cathode on electrochemical properties for lithium polymer batteries. Electrochim. Acta.

[195] Ren D., Feng X., Liu L., Hsu H., Lu L., Wang L., Ouyang M. (2021). Investigating the relationship between internal short circuit and thermal runaway of lithium-ion batteries under thermal abuse condition. Energy Storage Mater..

[196] Kaliapemural M., Dharanendrakumar M.S., Prasanna S., Abishek K.V., Chidambarami R.K., Adams S., Reddy M.V. (2021). Cause and mitigation of lithium-ion battery failure. Review.

[197] Lin X., Khosravinia K., Hu X., Li J., Lu W. (2021). Lithium plating mechanism, detection, and mitigation in lithium-ion batteries. Prog. Energy Combust. Sci..

[198] Münster P., Diehl M., Frerichs J.E., Börner M., Hansen M.R., Winter M., Niehoff P. (2021). Effect of Li plating during formation of lithium ion batteries on their cycling performance and thermal safety.

[199] Gao Z., Xie H., Yang X., Niu W., Li S., Chen S. (2022).

[200] Koleti U.R., Bui T.N., Dinh T.Q., Marco J. (2021). The development of optimal charging protocols for lithium-ion batteries to reduce lithium plating. J. Energy Storage.

[201] Zhu Y., Xie J., Pei A., Liu B., Wu Y., Lin D., al., e. (2019).

[202] Wang H., Zhu Y., Kim S., Pei A., Li Y., boyle D., Cui Y. (2020). Underpotential lithium plating on graphite anodes caused by temperature heterogeneity.

[203] Redondo-Iglesias E., Venet P., Pelissier S. (2019). Efficiency degradation model of lithium-ion batteries for electric vehicles.

[204] Habib A., Hasan M., Issa G., Singh D., Islam S., Ghazal T. (2023). Lithium-ion battery management system for electric vehicles. Constraints, Challenges, and Recommendations..

[205] Sevugan P.A., Pradeep M., Krishnaswamy A., Karunamurthy K. (2021). Battery thermal management system for electric vehicles using phase change materials.

